# Adaptive NKG2C^+^CD57^+^ Natural Killer Cell and Tim-3 Expression During Viral Infections

**DOI:** 10.3389/fimmu.2018.00686

**Published:** 2018-04-20

**Authors:** Hassen Kared, Serena Martelli, Shu Wen Tan, Yannick Simoni, Meng Li Chong, Siew Hwei Yap, Evan W. Newell, Sylvia L. F. Pender, Adeeba Kamarulzaman, Reena Rajasuriar, Anis Larbi

**Affiliations:** ^1^Singapore Immunology Network (SIgN), Aging and Immunity Program, Agency for Science Technology and Research (A*STAR), Singapore, Singapore; ^2^Academic Unit of Clinical and Experimental Sciences, Faculty of Medicine, University of Southampton, Southampton, United Kingdom; ^3^Centre of Excellence for Research in AIDS (CERiA), University of Malaya, Kuala Lumpur, Malaysia; ^4^Faculty of Medicine, University of Malaya, Kuala Lumpur, Malaysia; ^5^The Peter Doherty Institute for Infection and Immunity, University of Melbourne, Melbourne, VIC, Australia; ^6^Department of Microbiology, Yong Loo Lin School of Medicine, National University of Singapore, Singapore, Singapore; ^7^School of Biological Sciences, Nanyang Technological University, Singapore, Singapore

**Keywords:** natural killer cells, maturation, exhaustion, senescence, checkpoint blockade, aging, chronic infection, cancer

## Abstract

Repetitive stimulation by persistent pathogens such as human cytomegalovirus (HCMV) or human immunodeficiency virus (HIV) induces the differentiation of natural killer (NK) cells. This maturation pathway is characterized by the acquisition of phenotypic markers, CD2, CD57, and NKG2C, and effector functions—a process regulated by Tim-3 and orchestrated by a complex network of transcriptional factors, involving T-bet, Eomes, Zeb2, promyelocytic leukemia zinc finger protein, and Foxo3. Here, we show that persistent immune activation during chronic viral co-infections (HCMV, hepatitis C virus, and HIV) interferes with the functional phenotype of NK cells by modulating the Tim-3 pathway; a decrease in Tim-3 expression combined with the acquisition of inhibitory receptors skewed NK cells toward an exhausted and cytotoxic phenotype in an inflammatory environment during chronic HIV infection. A better understanding of the mechanisms underlying NK cell differentiation could aid the identification of new immunological targets for checkpoint blockade therapies in a manner that is relevant to chronic infection and cancer.

## Key Messages

- Acquisition of CD57 and NKG2C on adaptive NK cells is driven by HCMV and inflammation
- Tim-3 regulates the function of adaptive CD57^+^NKG2C^+^ NK cells
- Loss of Tim-3 in CD57^+^ NK cells marks cytotoxic-deficient NK cells during HIV infection.

## Introduction

As a key player of the innate immune system, natural killer (NK) cells provide vital protection against viruses and tumor cells during the acute and early stages of infection. The acquisition of effector functions during NK cell differentiation occurs *via* an intricate series of cellular and molecular events, orchestrated by specific transcription factors (TFs), such as T-bet (T-box transcription factor), Eomes (eomesodermin), Zeb2 (zinc finger E-box binding homeobox 2), and Foxo3 (forkhead box O3) (1)—ultimately generating mature cells that exhibit phenotypic signatures characterized by the expression of NKG2C (2), CD57 (3–5) and of activating killer immunoglobulin-like receptors (KIRs) (4). Among the listed TFs, Zeb2 is required for the terminal differentiation of NK cells (6), while Foxo TFs inhibit terminal NK cell development (7). These TFs direct changes in the expression of inhibitory or stimulatory molecules on NK cells, such as programmed cell death 1 (PD-1) (8), that subsequently modulate the immune response upon ligand binding. However, our understanding of the specific control that individual TFs have on NK cell function is limited at this stage.

A better understanding of the specific roles that individual transcriptional factors play in regulating the NK cell functions may help to elucidate the mechanisms involved in the modulation of NK cell maturation during viral infection and cancer, which is vital for pathogen clearance. Consequently, this may yield critical insights into the therapeutic implications of immune checkpoint blockade as a means to enhance NK cell activity within these disease contexts. With this goal in mind, we performed deep phenotyping of adaptive NK cells, particularly from human immunodeficiency virus (HIV) and human cytomegalovirus (HCMV)-infected donors, as these chronic infections have been implicated in driving the maturation and differentiation of NK cells (3, 5, 9, 10). Recent studies have linked certain combination of KIR and HLA class I alleles expression in HIV or hepatitis C virus (HCV) infected individuals with disease progression, but data on its influence at the genetic or transcriptional level are limited (11–14). Viremic HIV infected patients presented an inverted NKG2A/NKG2C ratio (15) and the expansion of adaptive non-conventional NK cells that lacked FcRγ expression (16). The former two NK cell subsets differ in terms of phenotype (CD57, NKG2A, and NKG2C) and response to highly active antiretroviral therapy (HAART). Adaptive NK cells also demonstrated more functionality than conventional NK cells, as reflected by an enhanced release of IFN-γ (17) combined with an increased antibody-dependent cellular cytotoxicity activity, which furthers their potential for broad antiviral responses against cells infected with HCMV, HIV or HSV-1 (16, 18). We analyzed, in particular, maturation-dependent changes in the TF expression of NK cells, with the assumption that this knowledge would provide clues to their functional implications, as inferred from the contemporaneous expression of surface markers that govern NK cell function during viral infections.

Due to its high expression on NK cells, our study focuses on identifying a novel role for T cell immunoglobulin domain and mucin domain protein 3 (Tim-3) in directing NK-cell behavior and maturation. Tim-3, one of the three members of the human Tim family (with Tim-1 and Tim-4), was initially described as a negative regulator of type 1 immunity during autoimmune diseases (19). This type I trans-membrane protein has been implicated in the activation or inhibition of immune responses (20, 21) depending on the recruitment of intracellular mediators such as Bat-3 (22) or Fyn (23) on its cytoplasmic tail. Tim-3 has many ligands including the versatile Galectin-9 (19, 24), phosphatidyl serine (with a lower affinity than Tim-1 and Tim-4), high mobility group protein B1 (HMGB1) (25), and the recently discovered Ceacam-1 (26). The functional implications of specific or combinatorial engagement of Tim-3 by its different ligands remain unknown. Since our understanding of the role of Tim-3 in NK cells is at its infancy, we made inferences from observations with T cells, where Ceacam-1 was recently identified as an important inhibitory ligand (26). Like PD-1, Tim-3 identifies dysfunctional T cells that have undergone repeated stimulation, and we hypothesize that it may also regulate antiviral innate immunity in NK cells. While Tim-3 was first identified as a Th1 marker, it is expressed by a range of immune cell types, including Th17 cells, dendritic cells, mast cells, or macrophages (27), and its highest expression is found on NK cells (28, 29). Although Tim-3-expressing NK cells are highly functional in terms of their cytotoxicity and cytokine-secretion capabilities (30), the engagement of Tim-3 by one of its ligands (e.g., Galectin-9) or the overexpression of Tim-3 in cancer or during persistent infection, as in hepatitis B virus (31) and HIV (32) infections, can restrict NK cell function. Dysfunctional NK cells from the blood of patients with metastatic melanoma have also revealed—in proportion to the donor’s clinical stage—an elevated expression of Tim-3; and more importantly, the subsequent blockade of Tim-3 restores NK cell cytotoxicity and cytokine production capacity (33).

Given its crucial role in mediating immune tolerance and its relationship with T cell exhaustion during chronic viral infection and cancer (34, 35), Tim-3 has begun to attract attention as a plausible target in cancer immunotherapy. In our study, we compare the phenotype and function of NK cell subsets using two disease models that drive NK cell dysfunction, namely HCMV and HIV infection (with or without HCV co-infection), and analyze changes in transcriptional regulation during the major stages of NK cell maturation. We demonstrate here that the expression of Tim-3 contributes to the fine regulation of Foxo3 and T-bet expression and that its expression is also important for adaptive NK cell function and maturation.

## Results

### CD57 and NKG2C Represent Hallmarks of NK Cell Maturation During HCMV Infection

**Figure 1:**
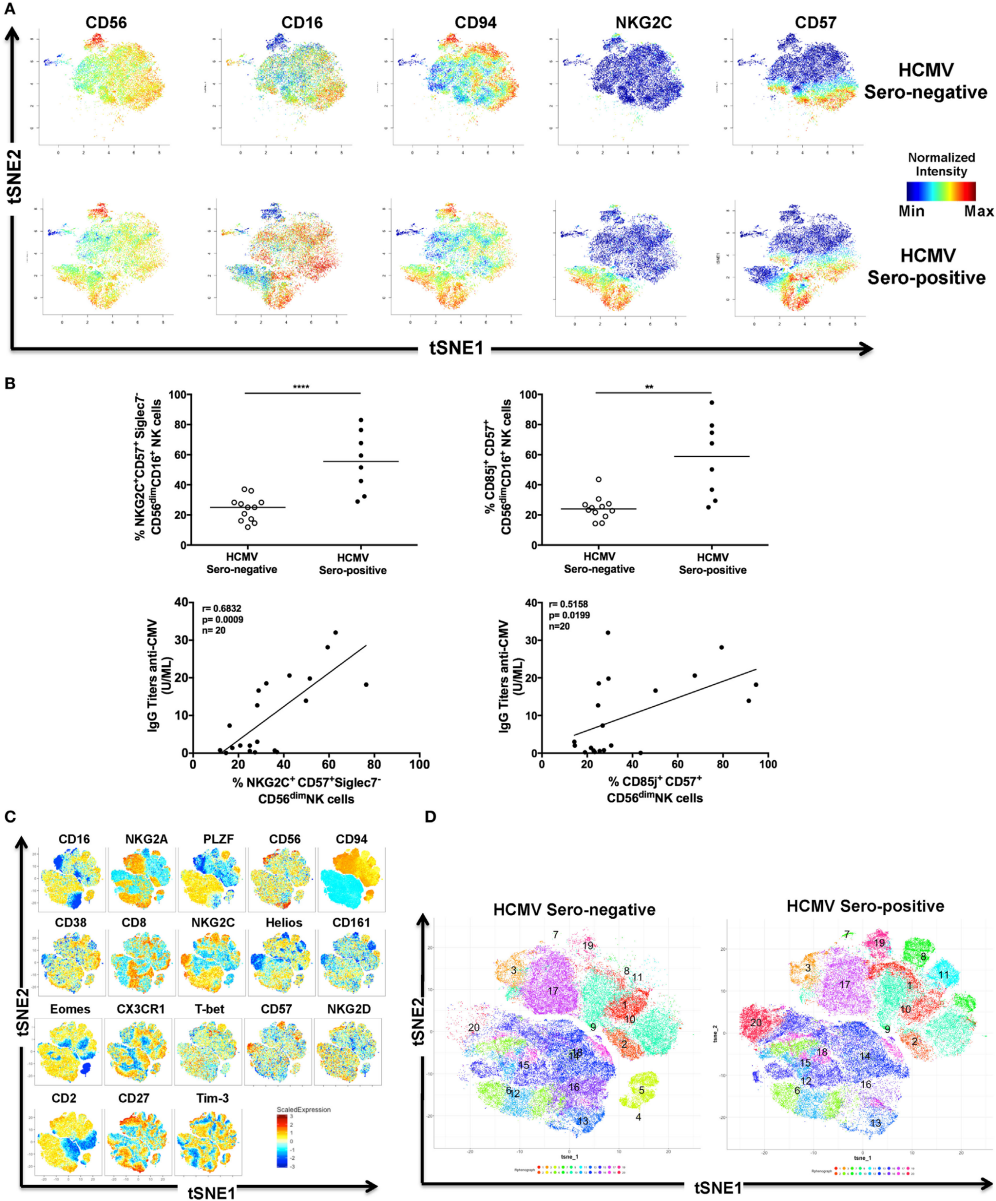

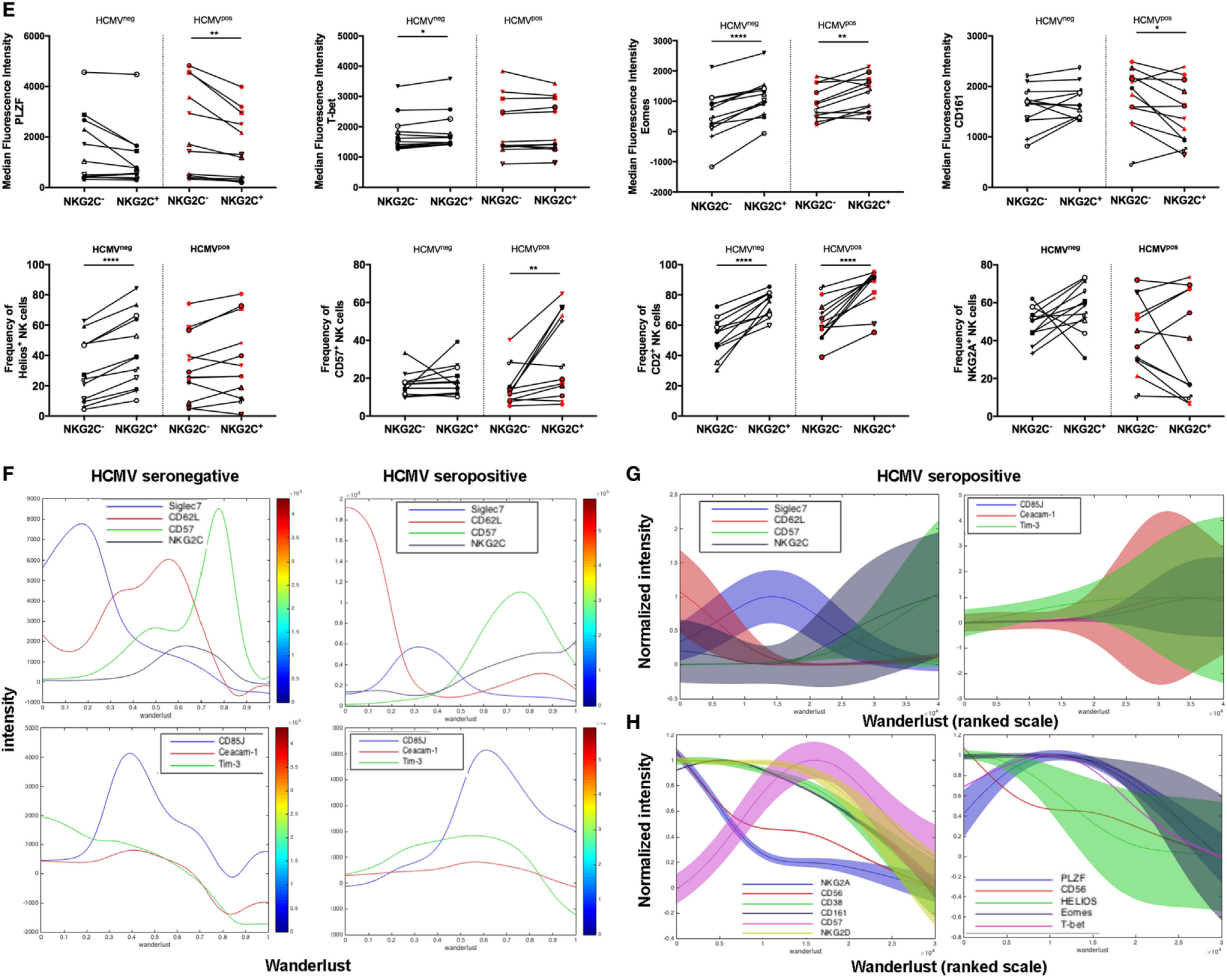
Identification of adaptive natural killer (NK) cells during human cytomegalovirus (HCMV) infection. **(A)** Identification of HCMV-induced human NK cells by mass cytometry. Frozen PBMCs from HCMV-seronegative and -seropositive donors were surface and intracellular stained for mass cytometry analysis. Samples were barcoded and acquired simultaneously (*n* = 6). *t*-Distributed stochastic neighbor embedding (tSNE) analysis of 23 parametric data was performed on live CD45^+^CD14^−^CD19^−^CD3^−^CD56^+^ NK cells from three HCMV-seropositive and three HCMV-seronegative donors. Event density in the tSNE field for all donors compiled together in which a same number of events per donor was included. Normalized protein expression levels for CD56, CD16, CD94, NKG2C, and CD57 in tSNE field were represented in red for high expression, whereas blue represents low expression (cold-to-hot heat map). **(B)** The antiviral response against HCMV drives acquisition of CD85j and NKG2C in CD57^+^ NK cells. The frequency and phenotype of NK cells subsets were analyzed by flow cytometry on freshly isolated PBMC (*n* = 28). The frequency of NKG2C^+^Siglec-7^−^ and CD85j^+^CD57^+^CD16^+^CD56^dim^ NK cells were positively correlated with the level of IgG antibodies specific to HCMV (*p* = 0.0009; *r* = 0.6832 and *p* = 0.0199; *r* = 0.5158, respectively). **(C)** Identification of human NKG2C^+^ adaptive NK cells by flow cytometry. PBMCs from HCMV-seronegative and -seropositive donors were surface and intracellular stained for flow cytometry analysis. Samples were acquired individually (*n* = 24). tSNE analysis of 18 parametric data was performed on live lymphocytes CD45^+^CD14^−^CD19^−^CD3^−^CD56^+^ NK cells from 12 HCMV-seropositive and 12 HCMV-seronegative donors. Event density in the tSNE field for all donors compiled together in which 5,000 events per donor were included. Normalized protein expression levels for single parameters in tSNE field were represented with a cold-to-hot heat map. **(D)** Identification of human NKG2C^+^ adaptive NK cells clusters during HCMV infection. HCMV-seropositive and -seronegative donors were compiled separately and analyzed for the repartition of clusters. **(E)** Phenotype of human NKG2C^+^ adaptive NK cells in presence or absence of HCMV infection. Gated NKG2C^+^CD56^dim^ and NKG2C^−^CD56^dim^ NK cells from 12 HCMV-seropositive and 12 HCMV-seronegative donors were analyzed by flow cytometry to detect specific signature of adaptive NK cells through the surface and intracellular expression of markers previously described as associated with HCMV infection. Each donor was identified by a unique symbol for the different molecules studied. The median fluorescent intensity or frequency of markers expression was represented for molecules with continuous or bi-modal expression, respectively. The median values were compared using a Wilcoxon matched-pairs signed rank test (*p < 0.05, **p < 0.01, ***p < 0.001, and ****p < 0.0001). **(F)** Characterization of human NK cell’s maturation according to Wanderlust trajectory. The Wanderlust trajectory is fixed to an arbitrary scale where the most immature NK cells (CD56^bright^) are at 0 and the most mature at 1. The traces demonstrated the relative expression patterns of Siglec-7, CD62L, CD57, NKG2C, CD85j, Ceacam-1, and Tim-3 across differentiation in HCMV-seropositive and HCMV-seronegative donors. **(G,H)** The expression of proteins and Wanderlust scale were normalized for the same markers than in panel **(F)** or for a new set of antigens such as NKG2A, NKG2D, CD38, CD161, and transcription factors promyelocytic leukemia zinc finger protein, Helios, Eomes, and T-bet. NKG2C and CD2 staining were included in the analysis but data are not shown. The variance of proteins expression was represented to illustrate heterogeneity between HCMV-seropositive donors.

Persistent viral infections can modify the phenotype and functions of NK cells (36). In attempting to understand the characteristics of “adaptive” NK cells induced by HCMV infection (37), we first performed a deep phenotyping of NK cells in healthy donors (*n* = 6) by mass cytometry [Cytometer time-of-flight mass spectrometer (CyTOF)]. With CyTOF, we were able to simultaneously study 35 parameters with single cell resolution (Figure 1A; Figure S1A and Table S1 in Supplementary Material). We used the *t*-distributed stochastic neighbor embedding (tSNE) algorithm as an initial analysis tool, which projects high dimensional data into two dimensional space (tSNE1 and tSNE2) through performing repeated pairwise comparison of randomly selected cellular phenotypes based on their marker expression—ultimately clustering closely related cells. The co-expression pattern of markers on NK cells generated specific clusters associated with cytokines receptors (CD122, CD127, and IL-18R), inhibitory receptors [killer cell lectin-like receptor G1 (KLRG1), CD160], TFs [promyelocytic leukemia zinc finger protein (PLZF), T-bet, and Eomes], cycling activity (Ki-67), granular contents (Granzyme A/B, Perforin), or adaptive NK cells (NKG2C, CD57).

To appreciate the phenotypical diversity of NK cells, we focused on identifying markers that associated with adaptive NK Cells. Since these cells could be induced by HCMV infection, we included HCMV-seropositive donors in our analysis. Donors were stratified according to their HCMV serostatus and classified as either HCMV-seropositive (*n* = 3) or HCMV-seronegative (*n* = 3), depending on their titers of HCMV-specific IgG antibodies (Figure 1A). Since the induction of NKG2C and CD57 expression on NK cells has been described during HCMV infection (4, 38), we identified and demarcated in the tSNE plot (Figure S1A in Supplementary Material), clusters associated with these markers to visualize the expression of other markers that associate with these HCMV-driven adaptive NK cells. The relatively low expression of NK cell receptors, cytokine receptors, and PLZF was combined with a strong expression of effectors molecules (Granzyme B and Perforin) and T-bet. The heterogeneous expression of some proteins, such as CD16, CD57, CD161, Eomes, or KLRG1, suggested a relative diversity of adaptive NK cells.

### Phenotypic Signature of HCMV-Induced NK Cells

We further validated the CyTOF HCMV-induced NK cell signature (Figure 1A; Figure S1A in Supplementary Material) by flow cytometry, using healthy donors who were identified either as HCMV-seropositive (*n* = 14) or HCMV-seronegative (*n* = 14), depending on their level of anti-HCMV IgG antibodies. As expected, the differential expression of molecules such as NKG2C (which may interact with viral protein UL40 on HCMV-infected cells) (39), CD85j (also called ILT2, which may recognize the viral protein UL18) (40), and Siglec-7 was observed between the two groups of donors, with HCMV-positive donors expressing more NKG2C and CD85j but less Siglec-7. Interestingly, positive correlations were also observed between CD57^+^ NK cells expressing NKG2C or CD85j and measurements of the anti-HCMV IgG titer in healthy donors (*n* = 20) (Figure 1B). Since the acquisition of adaptive phenotype by NK cells in seropositive donors appeared to be related to disease status, we sought to identify additional markers within the altogether heterogeneous group of adaptive NK cells to isolate specific subsets that were most representative of HCMV disease status (anti-HCMV IgM was not detected in the plasma of our Singaporean cohort suggesting the absence HCMV reactivation).

First, we sought to confirm the molecular signature of adaptive NK cells by CyTOF (37). The multi-parametric flow cytometry Symphony analyzer was used to compare NKG2C subsets in HCMV-seropositive or HCMV-seronegative group (*n* = 12 in each group) (Figures S1B,C in Supplementary Material). Total NK cells were downsampled to 5,000 events per donor, concatenated and analyzed by tSNE and the scaled intensity of each marker is represented in Figure 1C. The separation of healthy donors according to HCMV serostatus confirmed the existence of specific clusters that were enriched by HCMV, e.g., Cluster 20, which is characterized by the co-expression of CD57 and NKG2C (Figure 1D). In addition, there was a shift in the distribution of different NK cell populations—we observed an increased representation of clusters 8, 11, and 19 and reduced cellular numbers in clusters 4 and 5. The expression of NK receptors such as CD16, CD94, NKG2A, or NKG2C and maturation/inflammation markers such as CD161 or CD57 characterized these subsets (Figure S1D in Supplementary Material). Our analysis also reveals a specific molecular signature of NKG2C^+^ NK cells even in donors lacking an HCMV response (i.e., seronegative individuals, with a reduced frequency of NKG2C^+^ NK cells), characterized by an increased expression of CD2 and Eomes (Figure 1E). We further observed that the upregulation of CD2, Eomes, and CD57 were coupled with the downmodulation of CD161 and PLZF in adaptive NK cells from HCMV-seropositive individuals. The intensity (MFI) but not the frequency of Tim-3 expression was also significantly increased in HCMV-seropositive patients (data not shown), prompting us to investigate whether NK cells could also cis-express its putative ligand, Ceacam-1 (Figure S1E in Supplementary Material). When the cellular data of HCMV-positive donors were concatenated, we readily recognized the emergence of an adaptive population that was defined by the upregulation of CD57, NKG2C, Tim-3, and Ceacam-1 but were limited in Siglec-7 and CD62L expression (Figure S1F in Supplementary Material). Nevertheless, our understanding of the relationship between these markers and adaptive NK cells differentiation, as well as HCMV status, remained limited at this level of analysis.

### Adaptive NK Cell Differentiation and HCMV-Specific Immune Response

We sought to dissect the individual kinetics of each phenotypic marker during HCMV-driven cell differentiation with the Wanderlust software (41) (Figures 1F,G). To guide the analysis, CD56^bright^ NK cells were considered the most immature NK cell population (i.e., the starting population), and donors were segregated according to HCMV serological status to generate an unbiased model that reflects the maturation of NK cells in the absence or presence of HCMV infection *(*Figure 1F). To account for the heterogeneity between donors, the expression of each marker was normalized, and the variance was included in the graph for both HCMV-seropositive (Figure 1G) and HCMV-seronegative donors (Figure S1G in Supplementary Material). Among markers that were modulated by at least 50% of the maximum value that was detected among the different donors, we observed an early loss of CD62L coupled with the sustained upregulation of NKG2C in HCMV-seropositive donors (followed by the delayed loss of Siglec-7). The maturation of NK cells was also defined by the concomitant expression of CD57, CD85j, or NKG2C and a sustained expression of Tim-3 and Ceacam-1, although these relationships were only observed in HCMV-seropositive donors.

Next, we analyzed TF expression in seropositive and seronegative donors by Wanderlust. We extended this approach to other markers and reproduced the data independently of NKG2C to avoid limiting the study of adaptive NK cells to this specific antigen (Figure 1H) (*n* = 24 including 12 HCMV-seropositive donors). The downmodulation of CD56 from CD56^bright^ to CD56^dim^ NK cells was used as internal reference. Based on these parameters, we observed that the sequential loss of NKG2A, CD38, and CD161 is associated with the moderate or drastic increase in the expression of NKG2D and CD57, respectively. Despite observing a high degree of complexity within the evolution of TF kinetics, we were able to make two dominant observations: Helios was downmodulated earlier than the other TFs, and a decline in PLZF expression occurred almost simultaneously with an increase in CD57 expression. Since this analysis is limited by normalizing the data based on CD56^bright^ NK cells, which are the most immature but also the least frequent NK cell subset, we supported our model of progressive adaptive NK cell differentiation with a diffusion map analysis (Figure S1H in Supplementary Material), which also highlighted the upregulation of NKG2C, CD2, and Tim-3, and the downregulation of PLZF with adaptive phenotype acquisition.

Finally, the late divergence in the NK cell expression of CD57 and NKG2C may give rise to daughter cells that differentially express these markers. We identified four populations, based on the single or co-expression of CD57 and NKG2C (Figure S1I in Supplementary Material), and compared their Wanderlust score to determine their relationship with NK cell maturation (Figure S1J in Supplementary Material). Our data are indicative of NKG2C^−^CD57^−^ NK cells being the most immature NK subset, while NKG2C^+^CD57^+^ cells represent the most differentiated NK cell subset. In addition, our data demonstrate that NKG2C^−^CD57^+^ NK cells display a more differentiated phenotype than NKG2C^+^CD57^−^ NK cells.

### Regulation of CD57^+^NKG2C^+^ NK Cells Effector Polyfunctionality by Tim-3 Expression

**Figure 2:**
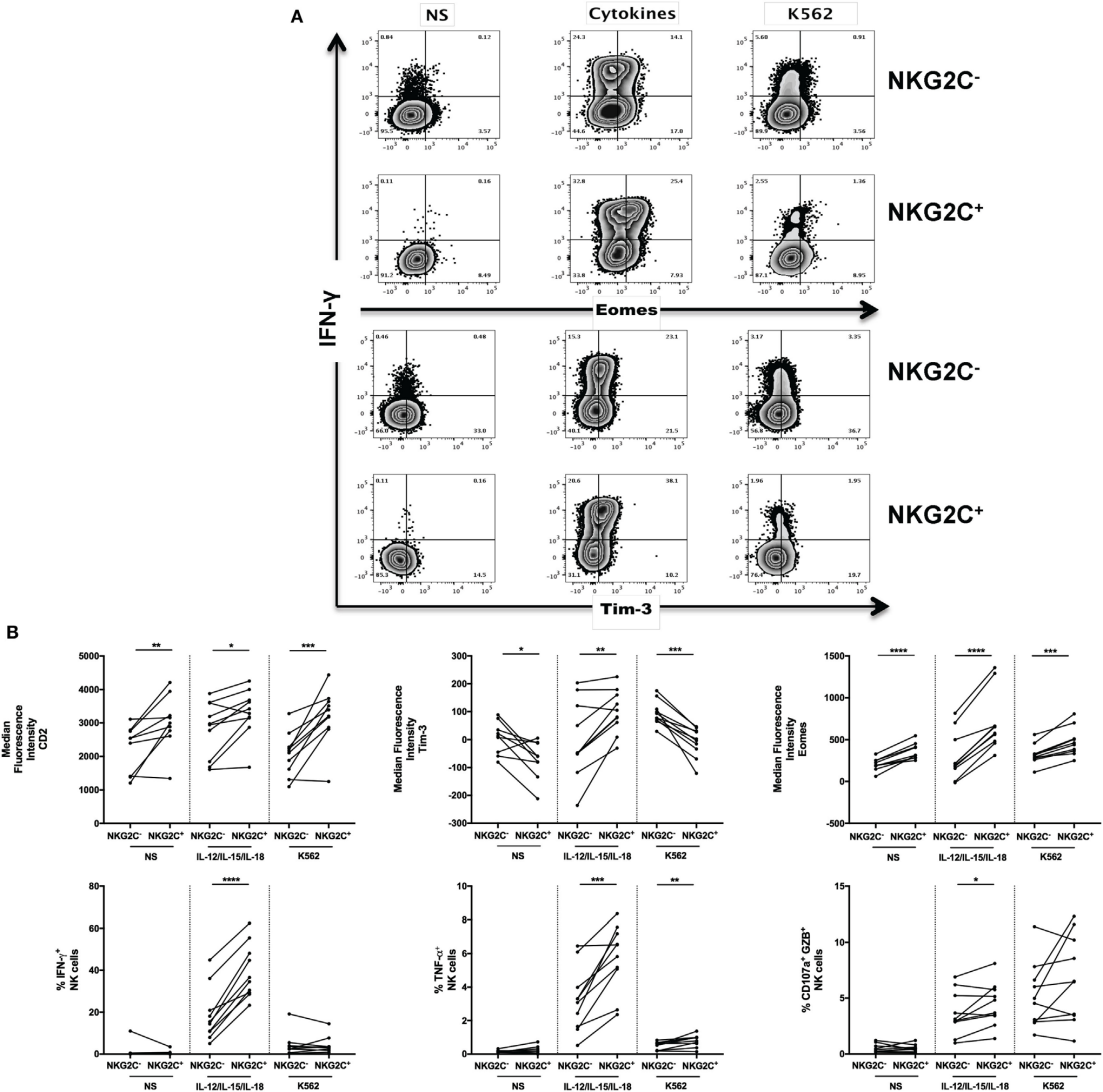

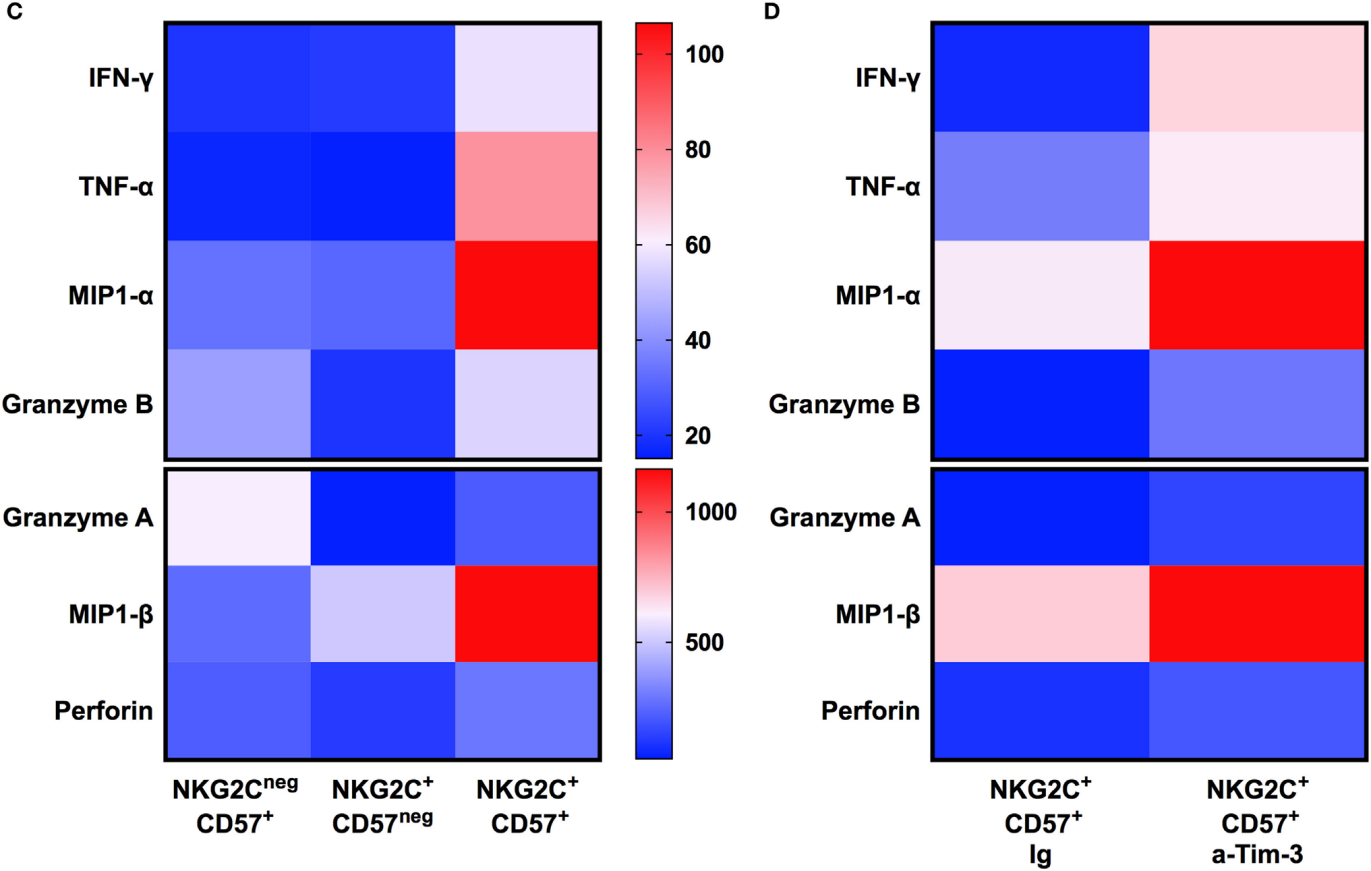

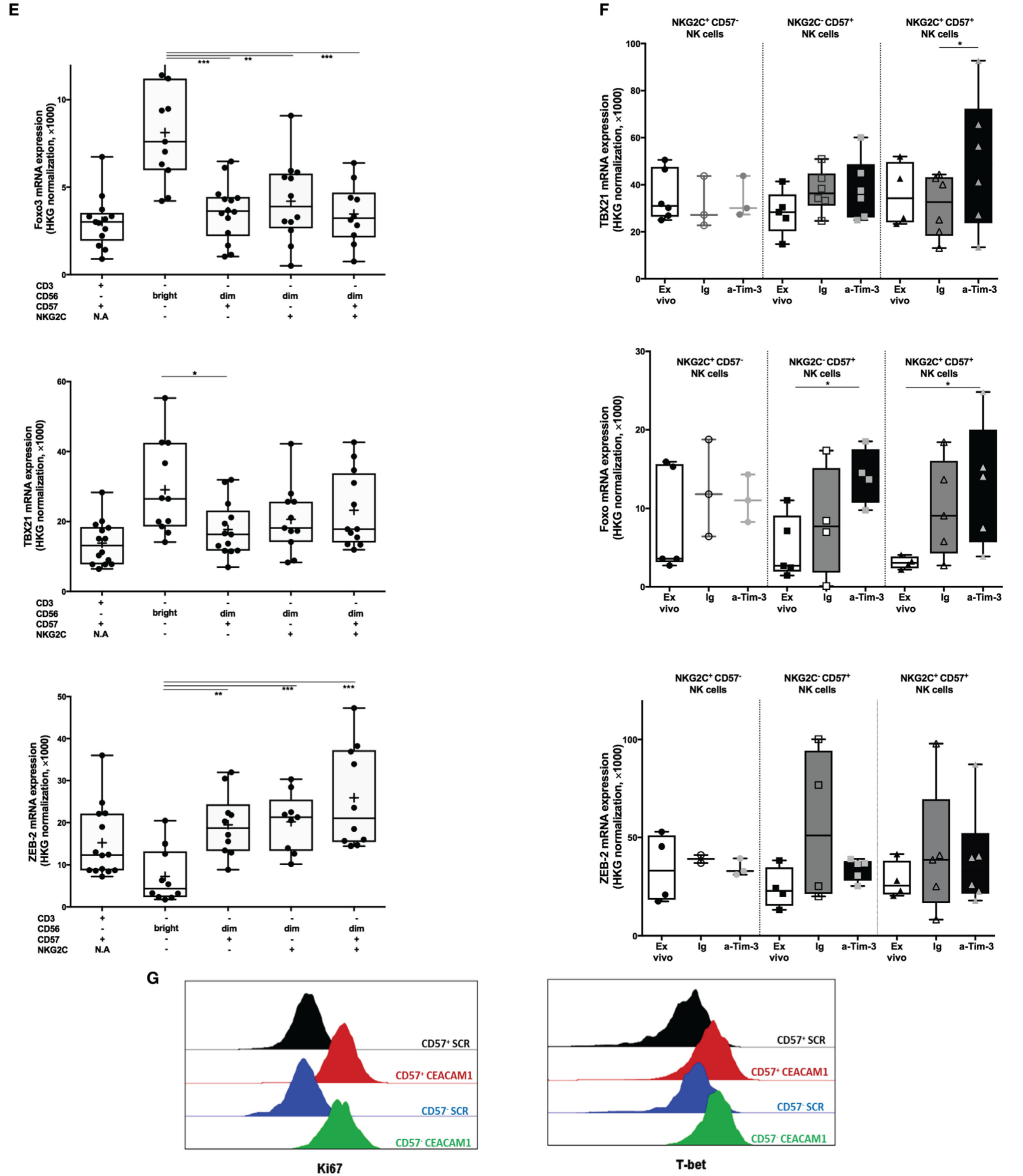

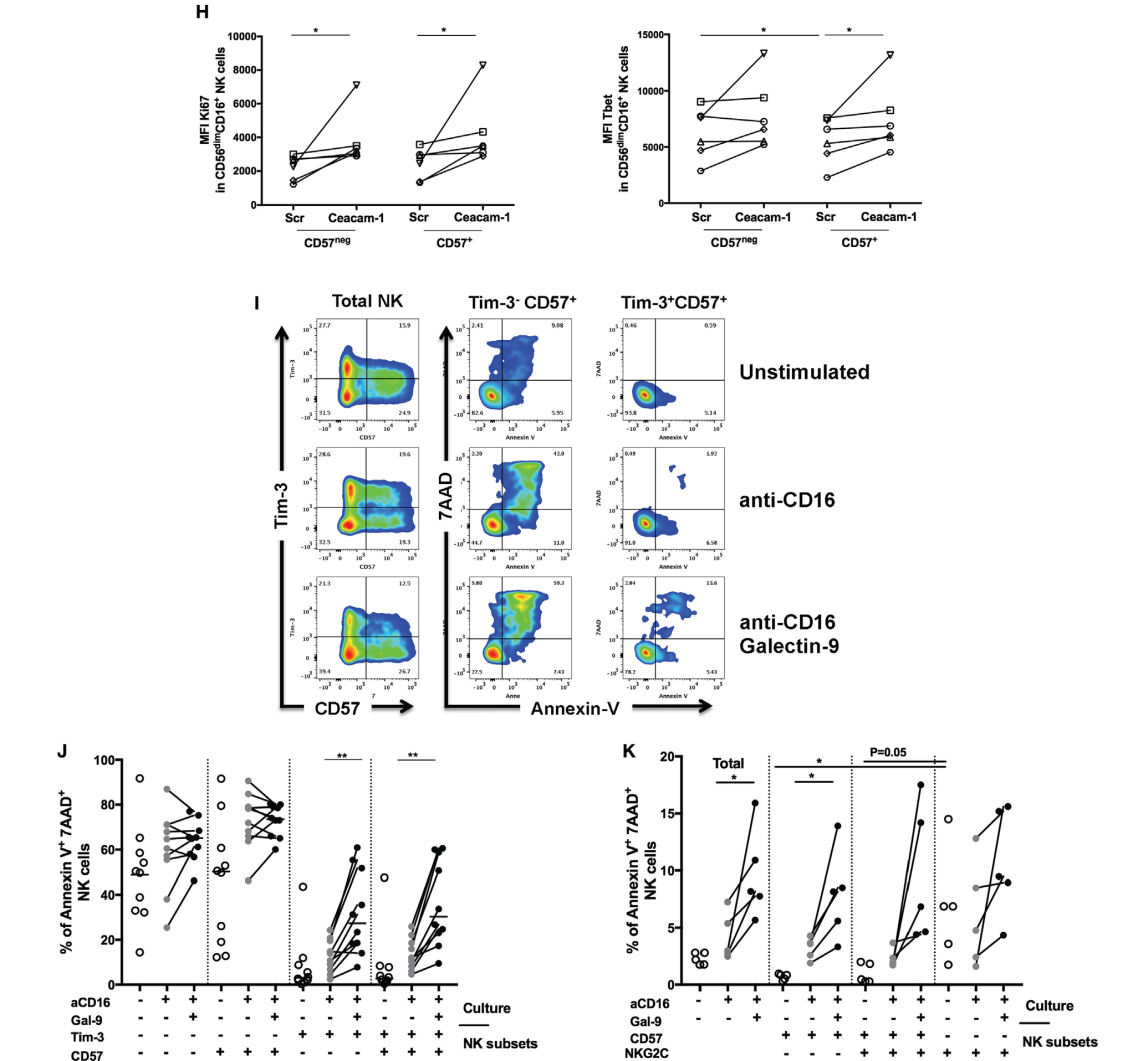
Regulation of adaptive natural killer (NK) cells by Tim-3 pathway during human cytomegalovirus (HCMV) infection. **(A)** Increased IFN-γ secretion is a hallmark of adaptive NK cells. Frozen PBMCs from 10 HCMV-seropositive donors were stimulated overnight with cytokines (IL-12/IL-15/IL-18) or by the contact of K562 cells. Surface and intracellular flow cytometry staining was performed to characterize the phenotype (Tim-3, Eomes) and functions (IFN-γ) of conventional (identified as CD45^+^CD14^−^CD19^−^CD3^−^CD56^+^NKG2C^−^) and adaptive (CD45^+^CD14^−^CD19^−^CD3^−^CD56^+^NKG2C^+^) live NK cells. **(B)** Increased IFN-γ secretion by adaptive NK cells is associated with enhancement of Tim-3 and Eomes expression. The median fluorescence intensity of CD2, Tim-3, and Eomes was measured in NKG2C^+^ NK cells before/after stimulation by inflammatory cytokines or interaction with cancer cell lines lacking MHC I expression (except Tim-3). The release of IFN-γ, TNF-α, and cytotoxicity (CD107a^+^GZB^+^) by gated NKG2C^−^ and NKG2C^+^ NK cells was assessed after stimulation with IL-12/IL-15/IL-18 (10/20/100 ng/ml, respectively) or interaction with K562 cell lines by flow cytometry. **(C)** Identification of polyfunctional NK cells using CD57 and NKG2C expression. CD56^dim^ NK cells from HCMV-seropositive donors were sorted according to CD57 and NKG2C expression and stimulated by CD16 crosslinking. Supernatants were collected and analyzed by Luminex. The heat map represented the median concentration of each molecule. **(D)** Regulation of CD57^+^NKG2C^+^ NK cells polyfunctionality by Tim-3 expression. Sorted NK cell subsets were preincubated with anti-Tim-3 (10 µg/ml) or IgG control before overnight stimulation by CD16 ligation. Supernatants of stimulated NK cells subsets were analyzed by Luminex, and the median concentrations of each analyte were represented by heat map. **(E)** Upregulation of Zeb2 expression in CD57^+^NKG2C^+^ NK cells and differential expression of Foxo3 and TBX21 during NK cell maturation. Sorted NK and T cell subsets from HCMV-seropositive donors were immediately lysed. Senescent T cells (CD57) and immature NK cells (CD56^bright^) were used as internal control. Transcription factor expression was analyzed by RT-PCR and normalized according to the expression of actin B used as a housekeeping gene. **(F)** T-bet and Foxo3 expression but not Zeb2 CD57^+^NKG2C^+^ NK cells are regulated by Tim-3 expression. Sorted NK cell subsets from HCMV-seropositive donors were preincubated with anti-Tim-3 or IgG control before overnight stimulation by CD16 ligation. Gene expressions of NK cell subsets were analyzed directly *ex vivo* and after *in vitro* stimulation with CD16 stimulation in presence of blocking Tim-3 antibody or IgG control. **(G)** Ceacam-1 silencing interferes with NK cell functions. Frozen PBMC from six HCMV-seropositive donors were pre-activated overnight with IL-2 and IL-15 before transfection with scrambled or specific Ceacam-1 siRNA. After resting, transfected cells were stimulated with CD16 crosslinking. CD57^+^ NK cells are depicted in black (Scr) or red (Ceacam-1) lines, and CD57^−^ NK cells are depicted in blue (Scr) or green (Ceacam-1) lines. The intensities of Ki-67 and T-bet were measured by intracellular staining on gated CD3^−^CD56^dim^CD16^+^CD57^−^ and CD3^−^CD56^dim^CD16^+^CD57^+^ NK cells. **(H)** Ceacam-1 silencing induces NK cell proliferation and T-bet expression. Ki-67 and T-bet expressions have been measured by flow cytometry after transfection with control scrambled (Scr) or specific Ceacam-1 siRNA. **(I)** Tim-3 expression regulates susceptibility of adaptive NK cells to apoptosis induced by Galectin-9. PBMCs of 10 HCMV-seropositive donors were stimulated by CD16 ligation in the presence of recombinant Galectin-9 (1 µM). Apoptosis was measured by the staining of annexin-V combined with 7-AAD staining on gated CD3^−^CD56^dim^CD16^+^ NK cells segregated according to expression of CD57, Tim-3 **(J)** or NKG2C **(K)**. Unstimulated cells and PBMCs incubated with Staurosporine were used, respectively, as a negative and positive control for apoptosis and necrosis. The mean values were compared using a paired *t*-test (*p < 0.05 and **p < 0.01).

As part of our strategy to decipher the functional implications of the molecular phenotype associated with adaptive NK cells, freshly isolated PBMCs were stimulated with CD16 crosslinking or K562 cells (which lack surface Major Histocompatibility Complex class I expression) to measure NK cell ADCC activity *ex vivo* (42) or induce an antitumor response, respectively (Figure S2A in Supplementary Material). Unstimulated PBMCs and PMA/Ionomycin stimulated PBMCs were, respectively, used to assess the spontaneous and absolute potential of NK cell activity. The secretory (IFN-γ and TNF-α) but not cytotoxic activity (degranulation marker CD107a combined with the expression of Granzyme B and Perforin) of gated CD57^+^ NK cells was enhanced in all the tested conditions (Figure S2A in Supplementary Material, *n* = 10). However, cytotoxicity was specifically heightened in CD57^+^ NK cells when a stronger dose of a-CD16 stimulation (10 µg/ml instead of 1 µg/ml, data not shown) was applied. Although the frequency of NK cells producing cytokines in response to PMA/Ionomycin was similar in both CD57 subsets by flow cytometry (Figure S2A in Supplementary Material, *n* = 12), the concentration of cytokines secreted and detected by Luminex was increased in CD57^+^ NK cells from HCMV-seropositive donors (Figure S2B in Supplementary Material, *n* = 6). However, as observed in Figure 1A, CD57 is also expressed on mature canonical NK cells and not only in adaptive NK cells. So, we measured the functional capacity of NKG2C^+^ and NKG2C^−^ NK cells from HCMV-seropositive donors in response to inflammatory cytokines (IL-12/IL-18 in presence of IL-15) or in response to reduced MHC class I presentation (Figure 2A). We assessed the secretion of cytokines (IFN-γ and TNF-α), degranulation (CD107a) and cytotoxicity (indirectly by the measurement of NK expressing CD107a^+^GZB^+^, CD107a^+^Perf^+^) and the phenotype of adaptive NK cells before and after stimulation (Figure 2B). Upon stimulation, NKG2C^+^ NK cells preserved their profile of heightened expression of CD2 (*p* = 0.011, *n* = 10) or Eomes (*p* < 0.0001, *n* = 10) and reduced PLZF expression (*p* = 0.05, *n* = 10). As previously described, Tim-3 was upregulated in adaptive NK cells and associated with IFN-γ production in response to IL-12/IL-15/IL-18 stimulation (*p* = 0.036, *n* = 10) (28). Although NKG2C^+^ NK cells were more reactive to cytokine stimulation than NKG2C^−^ NK cells in terms of their IFN-γ, TNF-α, and CD107a^+^GZB^+^ (*p* < 0.0001, *p* = 0.0005, and *p* = 0.0324, respectively; *n* = 10) response, we did not observe the same profile differences after incubation with K562 leukemia cells, as only TNF-α production was slightly increased in the latter situation (*p* = 0.0066, *n* = 10).

Finally, we sorted NK cells from HCMV-seropositive donors according to their CD57 and NKG2C expression (Figure 2C; Figure S2C in Supplementary Material) to further probe the molecular signature of these NK cell subsets. Analysis of the supernatants of these subsets stimulated by CD16 crosslinking revealed heterogeneous functions within the CD57^+^ NK cells that were associated with NKG2C expression, including differential release ability: MIP-1α/β, IFN-γ, TNF-α, Granzyme A/B, and Perforin (Figure 2C; Figure S2C in Supplementary Material). Overall, NKG2C^+^CD57^+^ NK cells were the most polyfunctional and secreted the highest quantities of MIP-1α/β, IFN-γ, TNF-α, Granzyme B, and Perforin.

The expression of Tim-3 in NK cells and the ascribed role of Tim-3 as a co-inhibitory molecule in T cells prompted us to investigate its relationship with NKG2C and CD57 expression in NK cells and its involvement in the modulation of NK cell function. NKG2C^+^CD57^−^, NKG2C^−^CD57^+^, and NKG2C^+^CD57^+^ NK cells from HCMV-seropositive participants were sorted, preincubated with neutralizing anti-Tim-3 or control IgG mAbs, and stimulated with CD16 crosslinking (Figure 2D); the preincubation step was necessary as Tim-3 neutralization was observed to be limited when the anti-Tim-3 antibody was added simultaneously with the sorted cells into CD16-coated wells (24). As Tim-3 blockade did not significantly change the secretory profile of NKG2C^+^CD57^−^ or NKG2C^−^CD57^+^ NK cells (Figure S2C in Supplementary Material), our future experiments were focused on NKG2C^+^CD57^+^ NK cells.

Tim-3 neutralization enhanced the functionality of NKG2C^+^CD57^+^ NK cells. Increased levels of TNF-α, MIP-1α/β, and Granzyme B were detected after Tim-3 blockade in the supernatants of stimulated sorted NK cells (*p* = 0.0313 for all the listed molecules; *p* = 0.0625 for Granzyme A and Perforin secretion, *n* = 6).

### Transcriptional Signature of CD57^+^NKG2C^+^ NK Cells Is Regulated by Tim-3/Ceacam-1 Expression

As the expression of effector molecules by NK cells are regulated by different TFs, we sought to determine whether adaptive NK cells had a unique genetic signature. We sorted and lysed immediately different NK subsets (CD56^bright^, conventional and adaptive NK cells) to measure the mRNA levels of NK-related TFs. Here, we observed the differential expression of TFs—Foxo3, T-bet, and Zeb2 between CD56^bright^ NK cells and others NK cell subsets (Figure 2E). Consistent with the Wanderlust data, the respective elevation and reduction of Zeb2 and Foxo3 mRNA in this subset of HCMV-induced NK cells, relative to the other NK cell subsets, is representative of a phenotype that is most advanced in term of development and function. We sought also to determine whether the coupling of Tim-3 neutralization with CD16 crosslinking might lead to any significant changes in the expression of other TFs and to ascertain whether these changes were coupled with the observed modulations in the levels of cytokines, chemokines, and cytotoxic molecules. Interestingly, Foxo3 expression was significantly increased after Tim-3 neutralization in NKG2C^+^CD57^+^ NK cells (*p* = 0.0159, *n* = 6). Moreover, Tbx21 mRNA (coding for T-bet) was upregulated in NKG2C^+^CD57^+^ NK cells following Tim-3 blockade (*p* = 0.0313, *n* = 6) (Figure 2F). These data suggest that Tim-3 may directly influence expression of Foxo3 and T-bet, but not Zeb2, in adaptive NK cells.

At this stage of investigation, the regulation of NK cell function by Tim-3 remains speculative. To further pursue this line of enquiry, we targeted Ceacam-1, which has recently been shown to be responsible for Tim-3-mediated exhaustion in T cells (26). Moreover, we have described in this study the co-expression of Tim-3 and Ceacam-1 by NK cell populations in HCMV-infected donors (Figures S1D,E in Supplementary Material). The role of Ceacam-1 in maintaining the Tim-3 mediated exhaustion of NK cells from HCMV-seropositive individuals (based on titer of anti-HCMV IgG) was evaluated by siRNA-mediated silencing (Figures 2G,H). The knockdown efficiency of *Ceacam-1* was evaluated by RT-PCR and compared with GAPDH silencing. The remaining gene expression of GAPDH and Ceacam-1 was elevated in the absence of cytokine pre-activation (Figure S2D in Supplementary Material); NK cells were thus stimulated with cytokines (IL-2/IL-15) before they were transfected with siRNA. Our first goal was to evaluate whether the silencing of Ceacam-1 expression could modulate degranulation, cytotoxicity, and cytokine production; and we did not observe any significant modulations in these aspects. The use of a modified protocol to orchestrate RNA interference may have led to the masking of any potential differences. Nevertheless, we investigated whether others functions and T-bet expression could be affected by the interruption of Ceacam-1 expression. Despite limited *Ceacam-1* silencing (75%), NK cell proliferation potential, as measured by Ki-67 expression, was restored (*p* = 0.0313 for CD57^−^ and CD57^+^ NK cells, *n* = 6) by siRNA transfection (Figure 2H). Whether or not the pre-activation procedure may universally induce Ceacam-1 (43) or Tim-3 acquisition on all other NK subsets; and whether Ceacam-1 may regulate all Tim-3 expressing NK cells (independently of CD57 expression) warrants further exploration. However, T-bet expression was only increased in CD57^+^ NK cells (*p* = 0.0313, *n* = 6), suggesting that the ability of Ceacam-1 to limit T-bet expression is most pronounced in CD57^+^ NK cells. Unfortunately, we were limited by the availability of conjugated NKG2C antibody and were unable to incorporate the detection of NKG2C in this panel, which is crucial for identifying adaptive NK cells.

Next, we sought to determine whether activation of the Tim-3 pathway could indeed limit NK cell function, including its cytolytic and proliferative potential. We first verified the ability of Galectin-9 (24) to trigger the Tim-3 pathway in NK cells (directly or otherwise); the addition of Gal-9 in the presence of anti-CD16 stimulation was observed to reduce NK cell proliferation (*p* = 0.0039) and T-bet expression (*p* = 0.002) in CD57^−^ NK cells (Figure S2E in Supplementary Material). In CD57^+^ NK cell, Gal-9 induced a reduction of T-bet (*p* = 0.084, *n* = 10) and cytokine production (*p* = 0.0078) in response to CD16 crosslinking (Figures S2E,F in Supplementary Material, respectively), although NK cytotoxicity was found to be preserved (data not shown). We were eager to determine whether the Gal-9 induced loss of NK cell function was due to the induction of apoptosis (Figure 2I). The addition of Galectin-9 in the presence of anti-CD16 stimulation reduced Tim-3 surface expression and therefore led to an increased frequency of Tim-3^−^ NK cells. From our data, we also observed that the expression of Tim-3 could predispose NK cells toward the induction of apoptosis in the presence of Gal-9 independently of CD57 expression (Figure 2J). The spontaneous cell death rate of NKG2C^+^CD57^−^ NK cells (no stimulation) appeared to be highest, but NKG2C^−^CD57^+^ NK cells were significantly more prone to apoptosis once stimulated *via* CD16 and Tim-3 (*p* = 0.0362, Figure 2K). Tim-3, but not NKG2C expression, was thus a more reliable indicator of apoptosis-prone NK cells. In summary, Tim-3 expression within adaptive NK cells may contribute to the loss of NK cell function, independently of cell maturation status, *via* the induction of apoptosis. Our data are consistent with a model where Tim-3 and Ceacam-1 neutralization induced T-bet expression in adaptive NK cells. Galectin-9 reduced T-bet expression and induced cell death of all Tim-3^+^ NK cells, independently of innate adaptive markers.

Finally, it was important to determine whether data from these *in vitro* analyses were recapitulated in clinical disease by testing the consistency of these findings—on the relationship between Tim-3, Ceacam-1, or T-bet expression and NK cell functions—in donors who suffered from chronic HIV and HIV/HCV infections and thus, experienced a heightened state of immune activation. Our analysis was based on both a longitudinal (*n* = 15, before and 48 weeks after the initiation of antiretroviral therapy) and cross-sectional (*n* = 103) cohort of HIV infected patients in comparison to healthy donors (*n* = 30) (Table S3 in Supplementary Material) (44). We stratified these HIV positive donors according to their age (Young, Middle age, Old), HCMV and HCV serostatus to examine the effects of HIV infection—with or without ongoing HCMV and/or HCV co-infection—on the expression of maturation markers and inhibitory receptors on NK cells (Figure S3A in Supplementary Material). It is important to observe that the smoking status of HCV-co-infected patients was different and may contribute to the increase of morbidities. The women were also overrepresented in the group of elderly people (HIV infected and non-infected) and may constitute a limit for the group comparison in the cross-sectional cohort.

### Inhibitory Receptor Expression on CD57^+^ NK Cells During HIV Therapy

**Figure 3:**
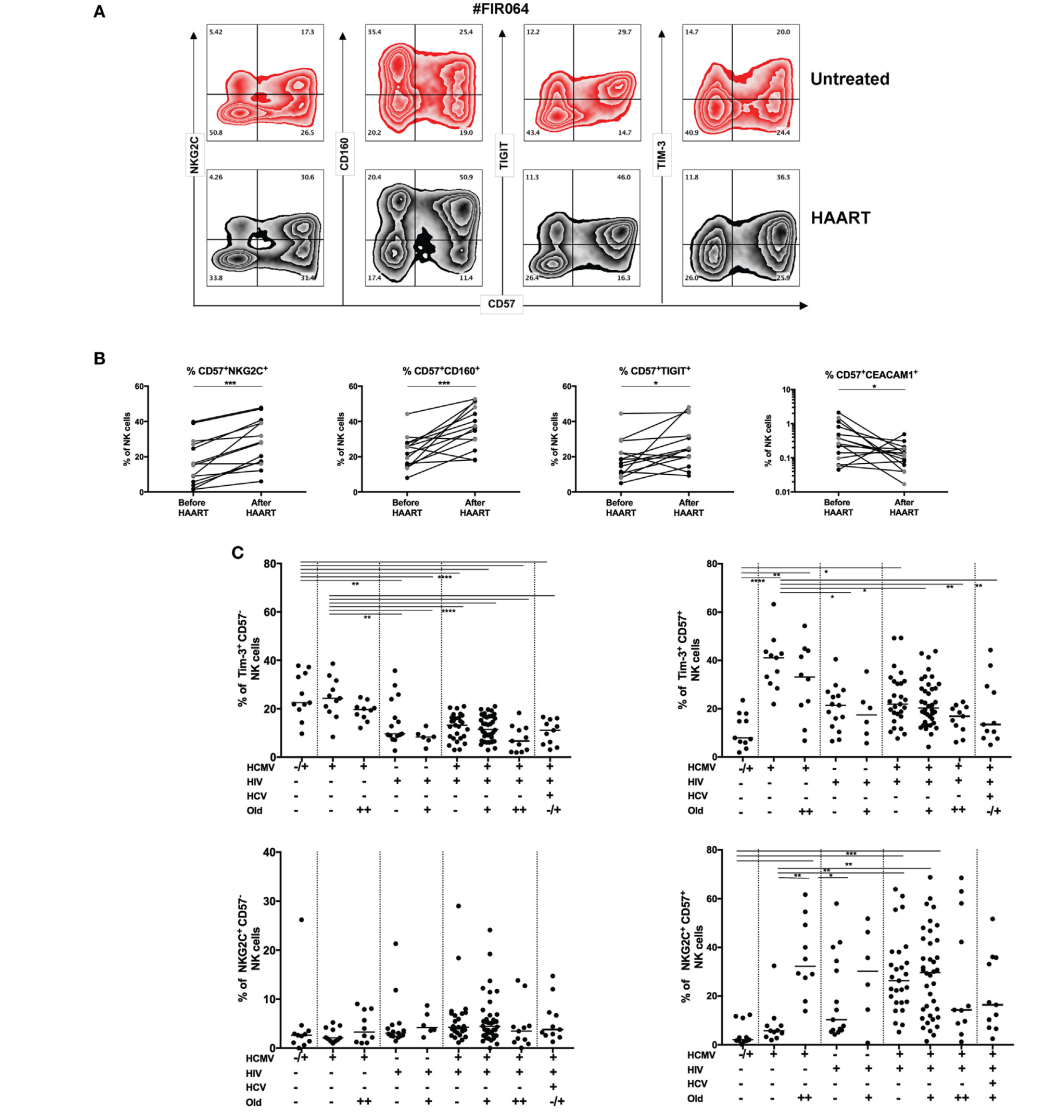

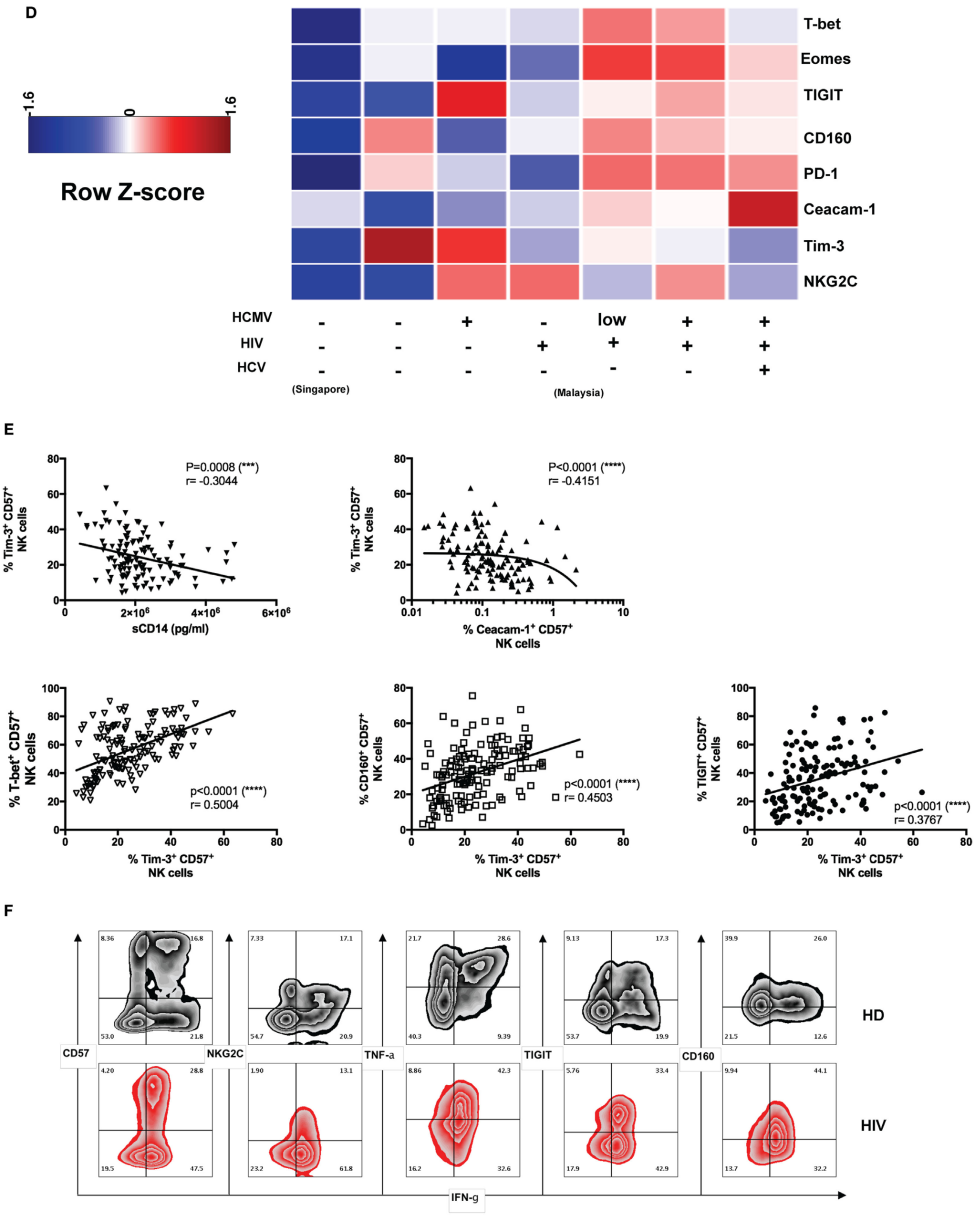

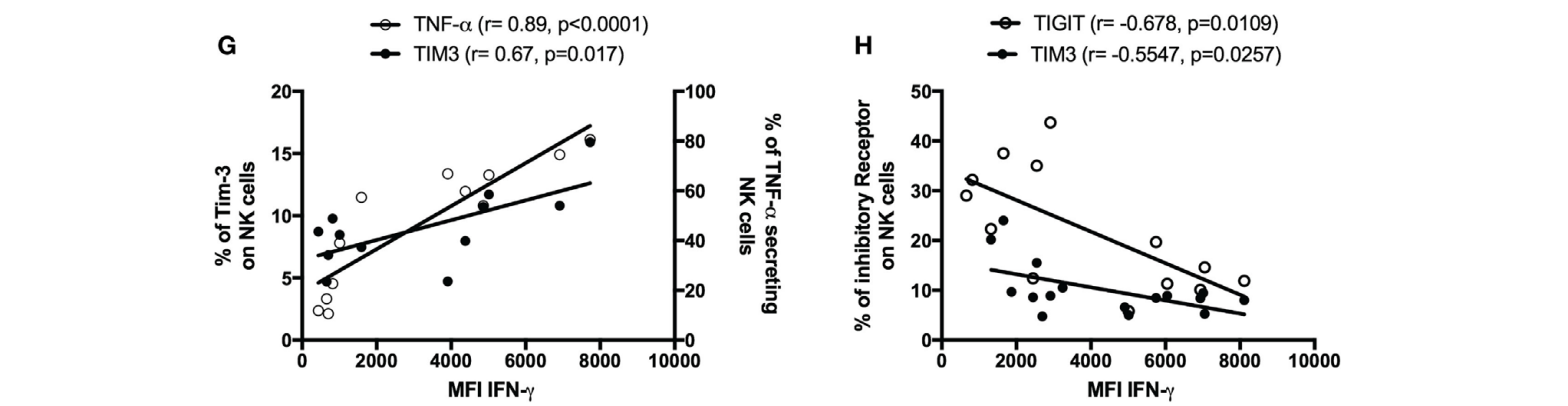
Tim-3 expression and adaptive natural killer (NK) cells during human immunodeficiency virus (HIV) infection. **(A)** Induction of CD57^+^NKG2C^+^ NK cells during HIV therapy. Untreated HIV patients were followed longitudinally before and after the initiation of antiretroviral treatment (48 weeks). Representative zebra plots of adaptive markers and inhibitory receptors expression on total NK cells (CD3^neg^CD56^+^) during highly active antiretroviral therapy (HAART) follow-up. **(B)** Inhibitory receptors expression of mature NK cells during HIV infection. Fifteen HIV infected patients were monitored before and after HIV treatment. Tim-3, T-bet, and Eomes modulation in NK cells were not statistically significant modulated by HAART. The median values were compared using a Wilcoxon matched-pairs signed rank test (*p < 0.05, **p < 0.01, ***p < 0.001, and ****p < 0.0001). **(C)** Downmodulation of Tim-3 but preservation of NKG2C on CD57^+^ NK cells during HIV infection. The frequencies of Tim-3 and NKG2C in CD57^−^ and CD57^+^NK cells (gated as viable CD56^+^CD3^neg^ cells) were studied in our cross-sectional HIV cohort (Malaysia) and compared with healthy donors from Singapore (first column) or Malaysia (second column). **(D)** Increased expression of inhibitory receptors and transcription factor (TF) expression in CD57^+^ NK cells during HIV infection. The frequencies of inhibitory receptors and TF in CD57^+^ NK cells (gated as viable CD56^+^CD3^neg^ cells) were normalized to obtain a mean row *Z*-score for each marker. A cold-to-hot heat map represented the relative expression of molecules in each patient group (age did not impact on the expression of most of these molecules and was not included here to simplify the representation). **(E)** Tim-3 expression, immune activation, and inhibitory receptors. Soluble CD14 correlated negatively with Tim-3 expression on mature CD57^+^ NK cells from HIV-infected and HIV-non-infected individuals (*n* = 119). The loss of Tim-3 expression is also coupled with acquisition of Ceacam-1 on CD57^+^ NK cells from HIV-infected and HIV-non-infected individuals (*n* = 128). The acquisition of Tim-3 was positively associated with T-bet (*n* = 145) and inhibitory receptors including CD160 (*n* = 145) and T-cell immuno-receptor with Ig and ITIM domain (TIGIT) (*n* = 145) in all donors. We used the non-parametric Spearman rank-order test to compare correlation between Tim-3 and sCD14 or proteins expression on CD57^+^ NK cells. We reported *r*-values and *p*-values. Analysis with *p < 0.05, **p < 0.01, and ***p < 0.001 were considered significantly different between the groups. **(F)** NK cells functions and inhibitory receptors expression during HIV infection. Representation of a combined phenotype and functional assay in total NK cells from non-infected and HIV infected patients. **(G)** Differential role of Tim-3 acquisition during NK cells activation in healthy donors or **(H)** during chronic HIV infection. Tim-3 or TNF-α secretion are positively correlated with IFN-γ intensity in NK cells from healthy donors. The inhibitory receptors TIGIT and Tim-3 are negatively correlated with IFN-γ secretion by NK cells in HAART-treated HIV patients.

Our first question was whether HIV infection and HAART influenced the imprinting of adaptive NK cells. Chronically infected HIV patients demonstrated higher frequencies of adaptive NKG2C^+^CD57^+^ NK cells posttreatment (Figures 3A,B). This NK cell subset was associated with the upregulated expression of exhaustion markers such as CD160 and T-cell immuno-receptor with Ig and ITIM domain (TIGIT), but downmodulation of Ceacam-1 in the absence of any significant differences in Tim-3 or T-bet expression (data not shown). Before HAART, the frequencies of adaptive NK cells in HIV positive donors were comparable to the percentages detected in the PBMCs from HIV uninfected but HCMV-seropositive donors (Figure 3C). After HAART, the frequencies of adaptive NKG2C^+^CD57^+^ NK cells were similar to those detected in HCMV/HIV infected donors (independently of age). The HCMV-mediated induction of NKG2C in treated patients with controlled HIV viremia is supported by observations from our cross-sectional study (Figures 3C,D) although this was not the case in donors who were co-infected with both HCV and HIV.

### Tim-3 Pathway and Immune Activation During HIV Infection

We sought to evaluate whether Tim-3 could modulate NK cell function during HIV infection. We were able to reproduce the data from recent studies, which described the loss of Tim-3 expression on NK cells during HIV infection (32, 45, 46). The decreased of Tim-3 expression in HIV infected patients was observed in gated CD57^−^ and CD57^+^ NK cells (Figure 3C). We observed a differential level of Tim-3 expression in our cohorts of healthy control between Singaporean (*n* = 11) and Malaysian donors (*n* = 30). Tim-3 was indeed more elevated in CD57^+^ NK cells in HIV non-infected participants from Malaysia. Despite the geographical proximity and similar ethnicities of participants, the socioeconomic status of these countries may have consequences on health status. We examined the hypothesis that differential levels of inflammation may be associated with Tim-3 expression. The level of inflammation in HIV infected patients was determined by measuring the levels of soluble CD14 and plasmatic CRP, as well as the kynurenin/tryptophan ratio [indoleamine-2,3-dioxygenase (IDO) activity] in serum. The systemic release of sCD14 constitutes a reliable marker of immune activation and reflects the extent of microbial translocation independent of the activation of monocytes and macrophages. The amount of circulatory sCD14 was increased by HCMV infection in both HIV uninfected (*p* = 0.02, *n* = 29) and HIV infected donors (*p* = 0.0487, *n* = 112) and by HIV infection in both HCMV-seronegative (*p* < 0.0001, *n* = 63) and seropositive donors (*p* = 0.0029, *n* = 78) (Figure S3B in Supplementary Material).

Immune activation (measured by sCD14 levels) also correlated negatively with the expression of Tim-3 on CD57^+^ NK cells (*p* = 0.0008, *r* = −0.3044, *n* = 119) (Figure 3E). Conversely, the levels of Ceacam-1 gradually increased with additional layers of concomitant infection (Figure S3A in Supplementary Material) and correlated negatively with the frequency of Tim-3^+^CD57^+^ NK cells (*p* < 0.0001, *r* = −0.4151, *n* = 102) (Figure 3E). The limited expression of Ceacam-1 prompted us to investigate if the downmodulation of Tim-3 could be explained by the interaction with its ligand, Galectin-9. The systemic concentration of this lectin increased during HIV infection in HCMV-seropositive donors (and during HCMV infection in HIV patients) (Figure S3C in Supplementary Material) (32, 47–50). Tim-3 expression on CD57^+^ NK cells was thus negatively associated with the cis-expression of Ceacam-1 and coupled with the increased systemic concentrations of Galectin-9 and sCD14. As expected, the expression of other markers of immune activation was elevated during HIV infection and we observed a positive association between the concentrations of sCD14 and both systemic IDO activity (Figure S4D in Supplementary Material) and plasmatic CRP (Figure S4E in Supplementary Material) in HIV/HCMV co-infected patients; moreover, these relationships were absent in healthy donors.

The frequencies of CD57^+^ NK cells expressing NKG2C, T-bet, and Eomes were also higher in HCMV-seropositive patients, but this was heterogeneously observed among the different donors. However, we were able to establish positive correlation between Tim-3 expression and the frequencies of CD57^+^ NK cells expressing T-bet, CD160, and TIGIT (*n* = 145, *p* < 0.0001) (Figure 3E). Most strikingly, the phenotypes traditionally associated with exhaustion for NK cells was reflective of HCMV status in HIV/HCV co-infection (Figure S3F in Supplementary Material) as patients with elevated anti-HCMV IgG antibodies titers (=500 U/ml) presented lower frequencies of CD57^+^ NK cells that expressed Tim-3, NKG2C, T-bet, and Eomes. Further investigation (including functional assays in response to cytokines, CD16 crosslinking and sensitivity to loss of HLA class I) is needed to understand how the cumulative burden of co-infection and inflammation qualitatively and quantitatively shifts TF expression in NK cells and renders them refractory in different co-infection settings. The individual capacity of HCMV, HCV, or HIV infection to modulate the expression of specific TFs in NK cells may explain why NK cells remain dysfunctional in HIV infected donors and express high levels of inhibitory molecules (PD-1, CD160, and TIGIT) despite the seemingly successful implementation of HAART.

### Inhibitory Receptors Expression and NK Cells Functions During HIV Infection

Due to cells number limitation in HIV patients, we studied the potential functions of NK cells in response to PMA/Ionomycin stimulation. The analysis of cytokine secretion and cytotoxicity has revealed that NK cells from in HIV-1 donors maintain a cytotoxic phenotype (CD107a^+^GranzymeB^+^) but exhibit a diminished capacity to secrete IFN-γ and TNF-α [*p* = 0.0014 (*n* = 18)] (Figures S3G,H in Supplementary Material). Moreover, the implementation of HAART did not appear to reverse these effects. Although the recovery of CD4 T cells following 48 weeks of HAART was significant [*p* = 0.0005 (*n* = 15), Table S3 in Supplementary Material], peripheral CD4 T-cell counts remained low (CD4 count <500 cells/μl)—suggesting that HAART-experienced donors continue to accommodate persistent alterations in immune functions. Whether this dysfunction persists in individuals who achieve normalized thresholds (>500 cells/μl) while on ART should be further explored.

While we were tempted to speculate that the reduced cytokine-secretion capacity of NK cells in untreated HIV-1 donors may result chiefly from the increased expression of Tim-3 on NK cells, we considered the precariousness of such a position as we and others have confirmed a decline in Tim-3 expression during ongoing HIV-1 infection. Nevertheless, the persistence of Tim-3 downregulation despite HAART implementation (23), suggests that Tim-3 dysregulation may influence the lack of complete restoration in NK cell function after HIV treatment. In our attempt to develop on this hypothesis, we used a combination of markers associated with exhaustion and adaptive NK cells (CD57, NKG2C) and cytokine release to compare the phenotype of adaptive NK cells in HIV infected patients and healthy donors (Figure 3F; Figure S3I in Supplementary Material). We observed that TNF-α and Tim-3 expression were strongly correlated with IFN-γ secretion in NK cells from healthy donors (Figure 3G), but a negative correlation between Tim-3 or TIGIT expression and IFN-γ detection was revealed by NK cells from HAART-treated HIV infected patients who were also HCMV-seropositive (Figure 3H). The involvement of TIGIT in suppressing NK cell function is supported by the observation that a subset of IFN-γ^low^ NK cells, which secrete less IFN-γ and TNF-α during HIV infection, exhibit an increased expression of TIGIT.

The loss of Tim-3 on adaptive NK cells is thus associated with systemic inflammation and decreased functionality during HIV infection. We could speculate that this implies a recovery (or preservation) of Tim-3 may be beneficial for the innate immune system of these patients.

## Discussion

Acute and chronic viral infections, such as those involving HCMV, HIV-1 (51) or West-Nile virus (52), may induce adaptive NK cells. The latter express NKG2C and CD57 and are regulated by inhibitory molecules such as Tim-3, which limit the amount of tissue destruction that can result from their prolonged activation (53). Throughout our lifetime, we can speculate that successive infection with pathogens may induce a footprint on adaptive NK cells that result in greater NK cell diversity. However, such an increase in the heterogeneity of the NK cell repertoire has been associated with a higher susceptibility to HIV infection, reflecting an NK cell deficiency in the antiviral response (52). Likewise, similar impairments were observed when HCMV-infected individuals were vaccinated against Flu (54, 55). Adaptive NK cells may thus be less responsive to neo-infections, vaccination, or antitumor activity. Thus, immune history could be considered as integral to the education of innate cells (including both NK cells and monocytes) and can be defined as trained immunity; some groups have described this process to be governed by epigenetic modifications (17, 56). We investigated here the role of Tim-3 on adaptive NK cells during viral infections.

The phenotype of adaptive NK cells was rigorously examined in this manuscript and was observed to align with the robust expression of NKG2C, CD57, or CD2 (57); and the reduced expression of PLZF, as observed previously (37). The downmodulation of PLZF in NK cells has already been identified as a hallmark of epigenetic regulation, which further leads to the hyper methylation of genes that code for FcεRγ, SYK, and EAT2 (37). However, the phenotype of adaptive NK cells has been heterogeneously described in the literature. Results from NKG2C-deficient patients have revealed that CD2 (57) or KIR expression (4) could compensate the absence of this receptor. The expression of NKG2C by FcRγ^+^ NK cells renders also more complicated the comparison between studies. We detected a residual NKG2C^+^ NK cell population in HCMV-seronegative individuals. This result suggests that adaptive NK cells differentiation is plastic and therefore could also be HCMV independent (3, 5, 9, 10). Next, the analysis with Wanderlust enabled us to impartially and chronologically model *in silico* the acquisition of markers throughout NK cell differentiation—it demonstrated the order-specific acquisition of NKG2C, CD85j, Ceacam-1, and Tim-3 in HCMV-seropositive donors. We also found that CD57 and NKG2C expression was sufficient to discriminate late-stage cytotoxic NK cells that can be differentiated by their expression of Foxo3, T-bet, and Zeb2.

A clearer understanding of how adaptive NK cells are differentially exploited by the individual viruses may help to improve current therapies against these pathogens. Through the more benign setting of HCMV infection, we demonstrate that the maturation of adaptive NK cells and the execution of NK cell function can be modulated by the Tim-3 pathway as its engagement, whether directly through Ceacam-1 and Galectin-9 or indirectly after activation alters the adaptive phenotype of NK cells. We acknowledge the controversy on whether these molecules are *bona fide* ligands for Tim-3 (19, 24), but since the aim of our experiment was to drive Tim-3 activity, stimulation *via* these pathways allowed us to obtain critical insight into the involvement of Tim-3 in modulating adaptive NK cells.

Hence, the identification of factors that drive Tim-3 expression on NK cells in different pathological contexts is useful for identifying targets that may drive NK cell function in specific disease settings. From the literature, we know that cytokines such as IL-27 (44) and IFN-β (46) drive the expression of Tim-3 in T cells; it is worth exploring whether these molecules are likewise responsible for Tim-3 expression in NK cells, as they may serve as attractive targets to boost NK cell activity. At this juncture, we also require specific knowledge of the factors that drive adaptive NK cell expansion to progress in our ability to improve NK cell functionality in the treatment of chronic viral infections. Type 1 IFN (58) and IL-33 (39) may improve the capacity of NK cells to control HCMV as already observed for murine CMV infection.

The link between Tim-3 and T-bet was shown to be relevant in the mediation of T cell exhaustion during chronic infection and cancer (59, 60), a similar relationship has not been described for NK cells. Here, we show that the expression of Tim-3 is associated with the downregulation of T-bet in NK cells. While the induction of Tim-3 expression was mediated by STAT3–NFIL3 (61) and Zeb2 in T cells, data from our healthy donors from Singapore and Malaysian HIV cohort suggest that Eomes may have a non-dispensable role in inducing Tim-3 expression during NK cell activation and maturation. Eomes is a key TF for NK cells differentiation, and its regulation by Foxo3 during the differentiation of pathogenic murine Th1 cells have been described (62). Our data further show that the concerted regulation of the Tim-3, T-bet, and Eomes pathways plays a role in the development of adaptive NK cell. More importantly, these signaling pathways contribute to functional deficits (both effector functions and proliferation) in adaptive NK cells—as neutralization of Tim-3 in NKG2C^+^CD57^+^ NK cells stimulated the release of cytokines, chemokines, and cytotoxic molecules; while blockade of Ceacam-1, the putative ligand for Tim-3, in CD57^+^ NK cells led to diminished T-bet expression and proliferation potential but preserved cytotoxicity or cytokine-secretion capacity. Based on these observations, it is likely that other Tim-3 ligands, such as phosphatidyl serine (on apoptotic cells), Galectin-9 or HMGB1, may be involved in modulating NK cell function *via* Tim-3, particularly at the epigenetic level (27).

Although the relationship between Tim-3 and NK cell maturation was clear in HIV-1 uninfected donors, its relevance in driving NK cell exhaustion during HIV-1 infection is less pronounced—since NK cells appeared to lose Tim-3 expression following progressive HIV-1 infection. However, it is possible that HIV-1 may mediate the uncoupling of Tim-3 expression and NK cell maturation as an evolutionary mechanism to support HIV survival, either by dampening the potency of NK cell antiviral functions (i.e., by delaying NK cell maturation) or by increasing the levels of immune activation—since the latter is the main driver of HIV pathogenesis. This hypothesis is supported by a study, which shows that Tim-3 downregulation in NK cells is most pronounced in patients who responded poorly to HAART (23). Moreover, levels of microbial translocation (and monocytes/macrophages activation) were clearly correlated with the loss of Tim-3 on mature NK cells in our study. In the literature, sCD14 levels have also shown association with alterations in NK cell activation and antiviral capacity (63, 64), although others have not linked this phenomenon to Tim-3 (or others inhibitory receptors) expression. The peripheral release of sCD14 was also associated with increased CRP in the plasma and enhanced IDO activity in our HCMV/HIV–infected patients; IDO metabolizes tryptophan into l-kynurenine, which is able to directly inhibit NK-cell functions (4, 65, 66). Overall, our data suggest that higher Tim-3 expression in NK Cells may be a marker of better prognosis during HIV infection, since its downregulation was indicative of a deficient IFN-γ response that cannot be reversed by antiviral therapy (32, 46). The impact of HAART on adaptive NK cells remains contentious. Although NKG2A/NKG2C ratio was initially restored by treatment (15), recent studies have described the stability of non-conventional FcRγ^−^ NK cells (16, 67). Despite the same duration of treatment (24 months), this discrepancy could be explained by differences in how adaptive NK cells are defined in these two studies and also by the potential heterogeneity of adaptive NK cells. When patients were matched by age and HCMV status, an expansion of NKG2C^+^CD57^+^ NK cells was observed in untreated HIV infected patients, and the increase in this specific subset of adaptive NK cells was maintained or even enhanced after 12 months of follow-up treatment. Although CD4 T-cell counts and viral load were not correlated to the frequencies of NKG2C^+^CD57^+^ NK cells (or Tim-3 expression), our data are confounded by the implementation of antiretroviral treatment, which contributed to the partial recovery of donor CD4 T cell counts (median value of 308 cells/μl at 48 weeks post-initiation, Table S3 in Supplementary Material). Nevertheless, the improved CD4 T-cell recovery and control over HIV replication may not be associated with the complete restoration of NK cell immunity in our Malaysian cohort.

Since HIV progression may be partially related to the downregulation of Tim-3 in NK cells, future studies should explore the mechanisms responsible for this phenomenon. Possible mechanisms that drive Tim-3 downregulation include the prolonged engagement of Tim-3 with Galectin-9 (or Ceacam-1), as this have been observed to occur here *in vitro*. The increased concentration of Galectin-9 during HIV infection [and more generally during viral infections (68)] supports this hypothesis, where the inhibition of IFN-γ secretion and cytotoxicity occurs through a Tim-3 independent pathway [mediated by cell surface protein disulfide isomerase (69), 4-1-BB (70), or CD44 (71, 72)]. Galectin-9 has a versatile role in the regulation of immune responses during viral infection is versatile, and its engagement with different receptors—depending on the stage of viral infection and inflammatory environment—could contribute to the modulation of adaptive NK cell functions. Moreover, HIV may also limit Tim-3 expression through the expression of microRNA-155 (73). In addition to the direct involvement of HIV-1—the emergence of other opportunistic pathogens, such as Leishmania (74) or HCV infection could also participate in mediating the loss of Tim-3 expression. Indeed, patients co-infected with HCV/HIV presented the lowest frequencies of mature NK cells that lacked NKG2C, Tim-3, and T-bet expression but exhibited the highest expression of Ceacam-1. Although the Tim-3 pathway may be dysregulated in progressive HIV infection, our data suggest that other inhibitory receptors (CD160, TIGIT, and PD-1) (8, 27), which limit NK cell functions remained relatively intact in HIV-1 infected individuals. The expression of TIGIT, in particular, was indicative of impairment in the capacity of adaptive NK Cells to secrete IFN-γ and TNF-α.

The functions of NK subsets are thus finely regulated and dependent on the expression of Tim-3 and Ceacam-1 (both cis- and trans-expression); and can also be modulated by the presence of Galectin-9 in the systemic environment (as observed during HCMV and HIV infections). The preservation of functional innate immunity during chronic infections such as HCMV, HIV, and HCV, thus requires the preservation of Tim-3 expression coupled with the limited acquisition of exhaustion molecules such as Ceacam-1, TIGIT, CD160 in adaptive NK cells and control of Galectin-9 release (or others putative Tim-3 ligands expression). In this context, the neutralization of immune activation by dietary supplementation of specific prebiotics may be beneficial to preserving NK cell functionality. This promising strategy, as demonstrated in a pilot study, resulted in a boost in NK cell activity in untreated HIV infected patients (75).

## Materials and Methods

### Ethics Statement

The study has been approved by the Ethics Committee of the NUS-IRB 09-256. Healthy adult donors were recruited at the National University of Singapore.

The HIV study was approved by the hospital institutional review board (MEC 896.32). All study participants provided informed written consent. Only adult donors were included in this work.

### Donors and Sample Preparation

Blood was collected from healthy adult donors at the National University of Singapore. Blood was collected from HIV infected and HIV non-infected individuals attending the University Malaya Medical Centre, Malaysia. Data on HIV-specific characteristics including HIV RNA, CD4 T-cell counts, antiretroviral drug history, and history of co-infections were obtained from patient medical records. Clinical characteristics of the patients are listed in Table S1 in Supplementary Material.

### Phenotyping

Cell phenotyping was performed by flow cytometry on 28 fresh PBMC samples from healthy donors. For each staining, 1 × 10^6^ PBMCs were used. Lymphocytes were gated based on live cells, FSC/SSC profile, and doublets/dead cell exclusion. T cells, B cells, and monocytes were excluded by CD3, CD19, and CD14 expression, respectively, followed by NK cell identification on CD3-negative lymphocytes using CD16 and CD56 expression and exclusion of double negative cells. The antibodies are listed in Table S2 in Supplementary Material. Flow cytometry was performed on an LSR Fortessa Cell Analyzer or a BD FACSYMPHONY (BD Biosciences), and automatic compensation was applied. Flow cytometry of HIV study was performed on a BD FACS Celesta (BD Biosciences) at University of Malaya, and automatic compensation was applied.

For CyTOF analysis, three million frozen PBMCs were stained as described previously (76). Data were acquired on a CyTOF2 instrument (DVS Sciences). The antibodies are listed in Table S1 in Supplementary Material.

### Flow Cytometry Functional Assay

Assessment of cytokine release and cytotoxic molecule degranulation was performed by flow cytometry on 20 PBMC samples from Healthy Donors. For each staining, 1 × 10^6^ PBMC were used. Cell stimulation with PMA/Ionomycin (50 ng/ml of PMA and 500 ng/ml of Ionomycin), with an anti-CD16 purified antibody-coated plate (1 and 10 µg/ml) or K562 cell line (at a 1:10 ratio) was performed. Total PBMCs were stimulated with the cocktail of cytokines (IL-12, IL-15, and IL-18) at the respective concentration of 10, 20, and 50 ng/ml. NK cells were gated as live lymphocytes CD3^−^CD19^−^CD14^−^CD56^+^ and studied according their expression of NKG2C.

Unstimulated and PMA/Ionomycin activated cells were used as negative and positive controls, respectively. Cells were incubated for 5 h at 37°C and 5% CO_2_ in the presence of CD107a antibody. Brefeldin A (eBioscience, final concentration 1×) and Monensin (eBioscience, final concentration 1×) were added during the final 4 h of incubation. For the list of antibodies used, refer to Table S2 in Supplementary Material. Flow cytometry was performed on an LSR Fortessa Cell Analyzer (BD Biosciences).

### HCMV ELISA

Frozen plasma samples were thawed and diluted 1:100 in the appropriate buffer. Seropositivity to HCMV was tested by ELISA (Genesis Diagnostics) according to the manufacturer’s instructions.

### sCD14 ELISA

Soluble CD14 was measured using the Quantikine sCD14 ELISA kit (R&D Systems, USA) according to the manufacturer’s instructions. Plasma samples were diluted 1:400 in the appropriate buffer and assayed in duplicate.

### Measurement of the Plasma Concentration of Tryptophan and l-Kynurenine

Plasma levels of tryptophan and l-kynurenine were directly analyzed by LC–MS/MS as previously reported (77).

### Multiplex Analyte Screening

Cell sorting was performed with a FACSAria III (BD Biosciences) on 12 samples according to CD57 and NKG2C expression. For the list of antibodies used for sorting, refer to Table S2 in Supplementary Material. After 18 h incubation with anti-CD16 (1 µg/ml), supernatants were collected and tested by Luminex assay. The Milliplex HCD8MAG-15K (Millipore) was used according to the manufacturer’s instructions and signal detected by Flexmap.

### Tim-3 Blockade

Tim-3 receptor blockade on NK cell functions was assessed by preincubating PBMCs or sorted NK cell subsets in the presence of anti-Tim-3 purified antibody (10 µg/ml) or Ig control (10 µg/ml) for 1 h before anti-CD16 stimulation (1 µg/ml; 18 h at 37°C and 5% CO_2_). For the list of antibodies used, refer to Table S2 in Supplementary Material.

### Quantitative Real-Time PCR

Natural killer cells sorted according to CD57 and NKG2C expression were lysed with RLT buffer with 1% of β-mercaptoethanol *ex vivo* or after 18 h anti-CD16 stimulation (1 µg/ml). RNA extraction was performed using an RNeasy Plus Micro kit (Qiagen) and reverse transcribed into cDNA using the SuperScript First Strand kit (Invitrogen). cDNA was analyzed by real-time PCR with the KAPA SYBR qPCR Master Mix kit (KAPA Biosystems) and the following primers from Qiagen: *ZEB2, TBX21*, and *FOXO3*.

### RNA-Mediated Interference

Seven frozen PBMC samples were electroporated with a Neon transfection kit and device (Invitrogen). Cells (3 × 10^5^) were incubated for 18 h with 10 ng/ml of IL-2 (Peprotech) and 10 ng/ml of IL-15 (Peprotech) and resuspended in 10 µl of buffer T (Neon kit, Invitrogen). *Ceacam-1* siRNA (Entrez Gene ID 634; detected transcripts NM_001024912.2, NM_001205344.1, and NM_001712.4) or negative control siRNA (at a final concentration of 100 nM; Ambion) were added to the cell suspension. Ten microliters of the suspension were electroporated (1,700 V, 20 ms, three pulses). GAPDH siRNA (Ambion) was used as a positive control to evaluate efficiency of the silencing. Cells were incubated for 24 h at 37°C and 5% CO_2_ and then stimulated in anti-CD16-coated plate (1 µg/ml) for 5 h at 37°C and 5% CO_2_ in the presence of CD107a antibody. Brefeldin A (eBioscience) and Monensin (eBioscience) were added during the last 4 h of incubation. Surface markers and functions by intracellular staining were assessed by flow cytometry as described earlier.

### Apoptosis Measurement

10 freshly isolated PBMCs were stimulated in anti-CD16-coated plate (1 µg/ml) for 5 h at 37°C and 5% CO_2_ in the presence or not of Galectin-9 (1 µM). Annexin-V and 7-AAD staining were performed according to the manufacturer’s instructions (BioLegend).

### Data Analysis

Flow cytometry data were analyzed using FlowJo (Treestar) and FACSDiva (BD Biosciences). Samples were compared using GraphPad Prism software (v.6.0c). Developmental trajectory during NK cell maturation was created with the Wanderlust algorithm (41). *tSNE analysis of Flow and Mass cytometry data*: unbiased representations of multiparameter flow cytometry data were obtained using the tSNE algorithm (78). The tSNE is a non-linear dimensionality reduction method that optimally locates cells with similar expression levels near to each other and cells with dissimilar expression levels further apart. tSNE analysis were performed using custom R scripts and Cytofkit software (79). *Statistical analysis*: groups of HCMV-seronegative and HCMV-seropositive donors were analyzed by Mann–Whitney *U* test to compare values. The Wilcoxon matched-pairs signed rank test was used for paired testing of median values of different subsets from the same donor. We used the non-parametric Spearman rank-order test to compare correlation between anti-HCMV IgG titer and frequency of NK cells subsets. We reported *r*-values and *p*-values. Analysis with *p < 0.05, **p < 0.01, and ***p < 0.001 was considered significantly different between the groups.

## Author Summary

Kared et al. demonstrate that highly differentiated NK cells expand upon chronic immune stimulation during persistent viral infection. Tim-3 regulates NK cells maturation through modulation of a transcriptional network that includes T-bet and Foxo3. Insights into this mechanism could pave the way to new immunotherapies and vaccinations.

## Ethics Statement

This study was carried out in accordance with the recommendations of Ethics Committee of the NUS-IRB 09-256 and by the hospital institutional review board (MEC 896.32) with written informed consent from all subjects. All subjects gave written informed consent in accordance with the Declaration of Helsinki. The protocol was approved by the Ethics Committee of the NUS-IRB 09-256 and by the hospital institutional review board (MEC 896.32).

## Author Contributions

Conceptualization: HK and AL; experimental work: HK, SM, ST; samples collection: HK, MC, YH, RR, AK, and AL; data analysis, HK, SM, YS, EN; writing—original draft: HK and SM; writing—review and editing: AL and SP; supervision: HK and AL; funding acquisition: AL, RR, and SP (VC scholarship and A*STAR ARAP).

## Conflict of Interest Statement

The authors declare that the research was conducted in the absence of any commercial or financial relationships that could be construed as a potential conflict of interest.

## Abbreviations

CyTOF, Cytometer time-of-flight mass spectrometer; HAART, highly active antiretroviral therapy; HBV, hepatitis B virus; HCMV, human cytomegalovirus; HCV, hepatitis C virus; HIV, human immunodeficiency virus; IDO, indoleamine-2,3-dioxygenase; KIRs, killer immunoglobulin-like receptors; KLRG1, killer cell lectin-like receptor G1; PD-1, programmed cell death 1; PLZF, promyelocytic leukemia zinc finger protein; TF, transcription factor; TIGIT, T-cell immuno-receptor with Ig and ITIM domain; Tim-3, T cell immunoglobulin domain and mucin domain protein 3.
