# Supplementary material for: Adaptive NKG2C+CD57+ Natural Killer Cell and Tim-3 Expression During Viral Infections
Source: Front Immunol. 2018 Apr 20;9:686. doi: 10.3389/fimmu.2018.00686 (PMC5919961; doi:10.3389/fimmu.2018.00686)
Supplement: Supplementary file 1 [file data_sheet_1.PDF]

Adaptive NKG2C$^+$ CD57$^+$ Natural Killer cell and Tim-3 expression during viral infections

A)

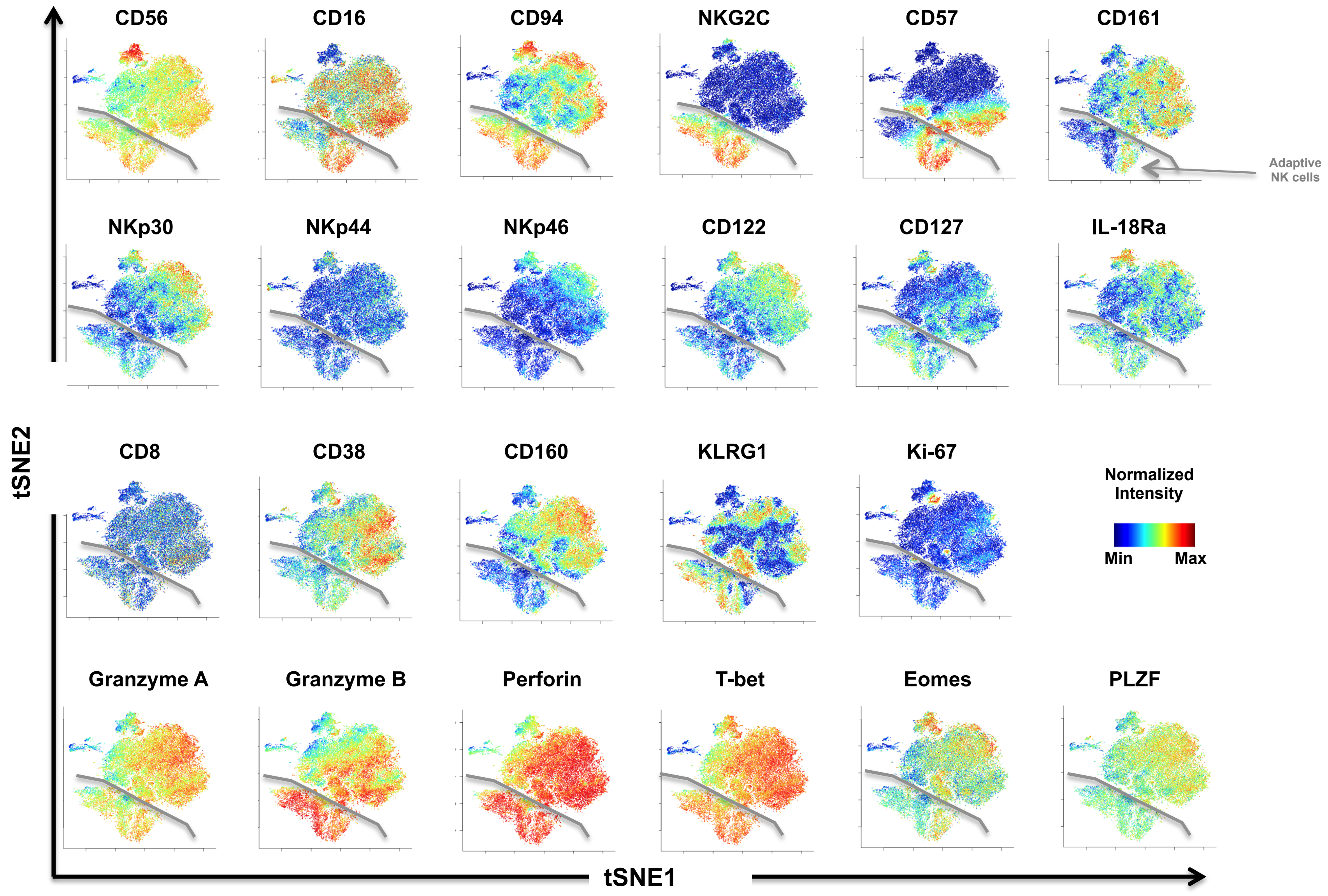
CD56
CD16
CD94
NKG2C
CD57
CD161
Adaptive NK cells
NKp30
NKp44
NKp46
CD122
CD127
IL-18Ra
CD8
CD38
CD160
KLRG1
Ki-67
Normalized Intensity
Min
Max
Granzyme A
Granzyme B
Perforin
T-bet
Eomes
PLZF
tSNE2
tSNE1

Figure S1

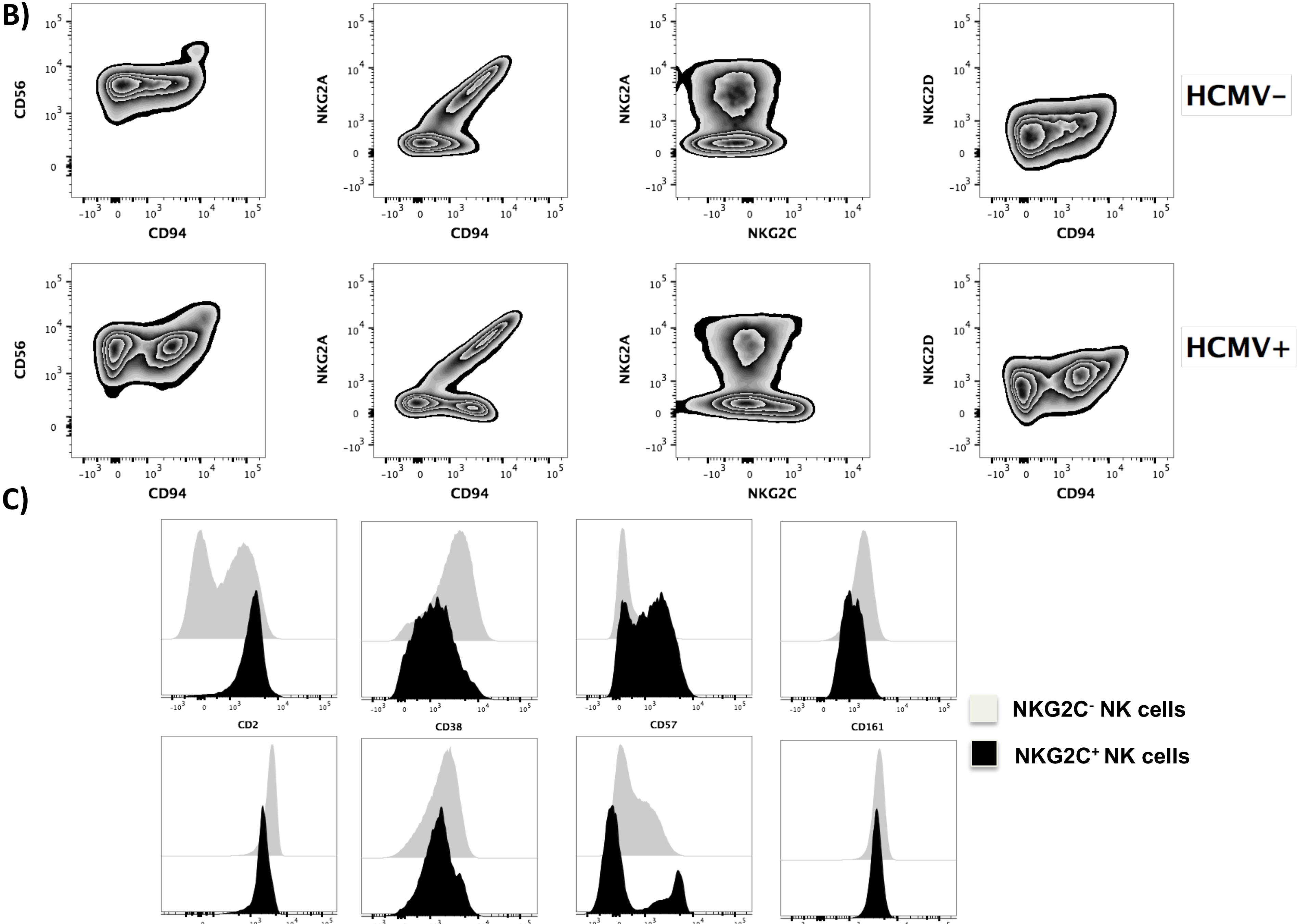
B)
CD56
CD94
NKG2A
NKG2C
NKG2D
HCMV−
HCMV+
C)
CD2
CD38
CD57
CD161
PLZF
Eomes
HELIOS
T-bet
NKG2C- NK cells
NKG2C+ NK cells

Figure S1

D)

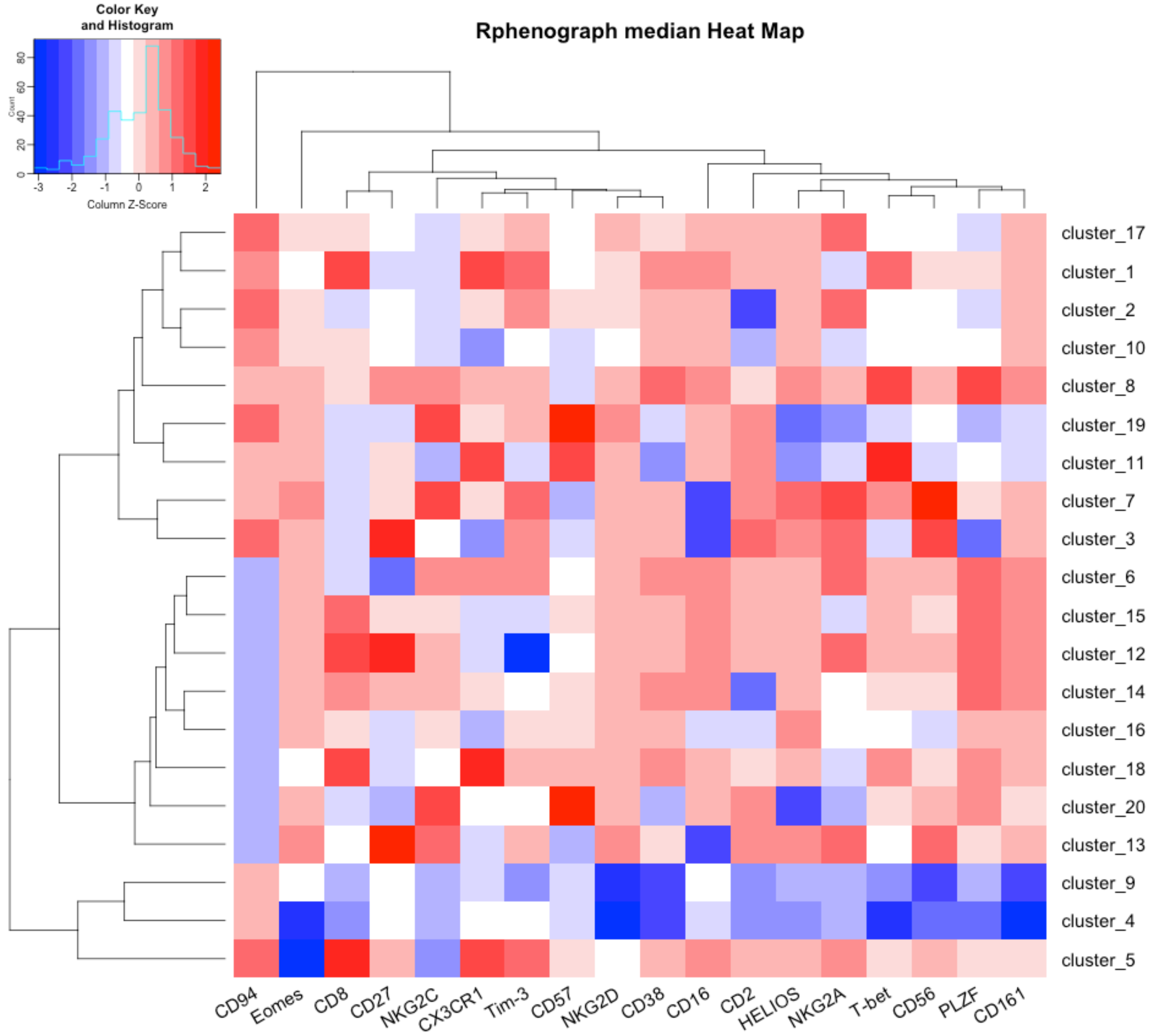
Color Key
and Histogram
Count
Column Z-Score
Rphenograph median Heat Map
cluster_17
cluster_1
cluster_2
cluster_10
cluster_8
cluster_19
cluster_11
cluster_7
cluster_3
cluster_6
cluster_15
cluster_12
cluster_14
cluster_16
cluster_18
cluster_20
cluster_13
cluster_9
cluster_4
cluster_5
CD94
Eomes
CD8
CD27
NKG2C
CX3CR1
Tim-3
CD57
NKG2D
CD38
CD16
CD2
HELIOS
NKG2A
T-bet
CD56
PLZF
CD161

E)

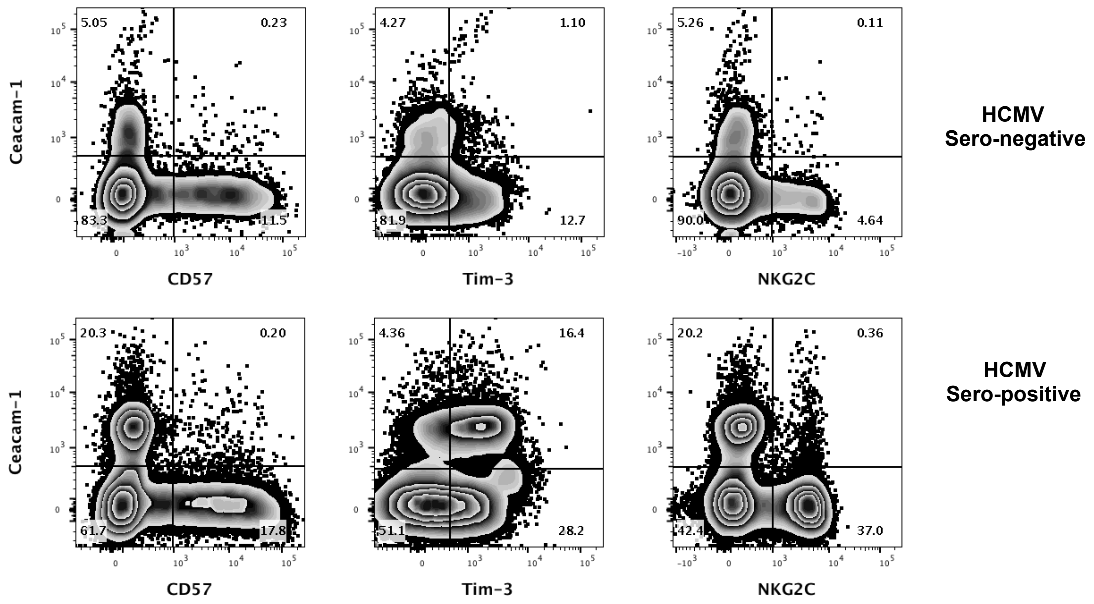

5.05
0.23
83.3
11.5
Ceacam-1
CD57
4.27
1.10
81.9
12.7
Tim-3
5.26
0.11
90.0
4.64
NKG2C
HCMV
Sero-negative
20.3
0.20
61.7
17.8
4.36
16.4
51.1
28.2
20.2
0.36
42.4
37.0
HCMV
Sero-positive

F)

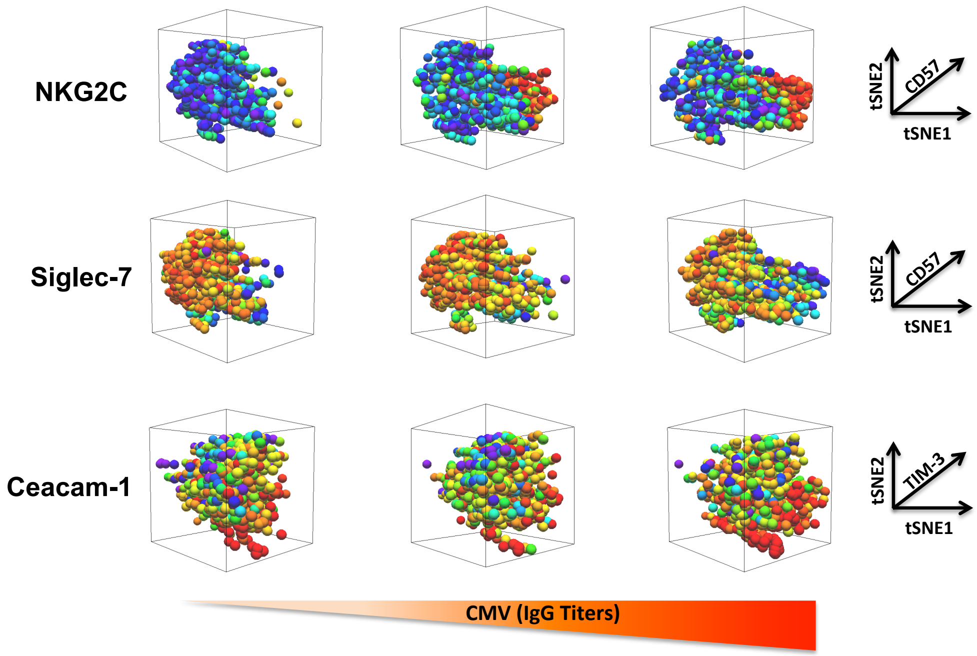

NKG2C
Siglec-7
Ceacam-1
tSNE2
CD57
tSNE1
TIM-3
CMV (IgG Titers)

Figure S1

G)

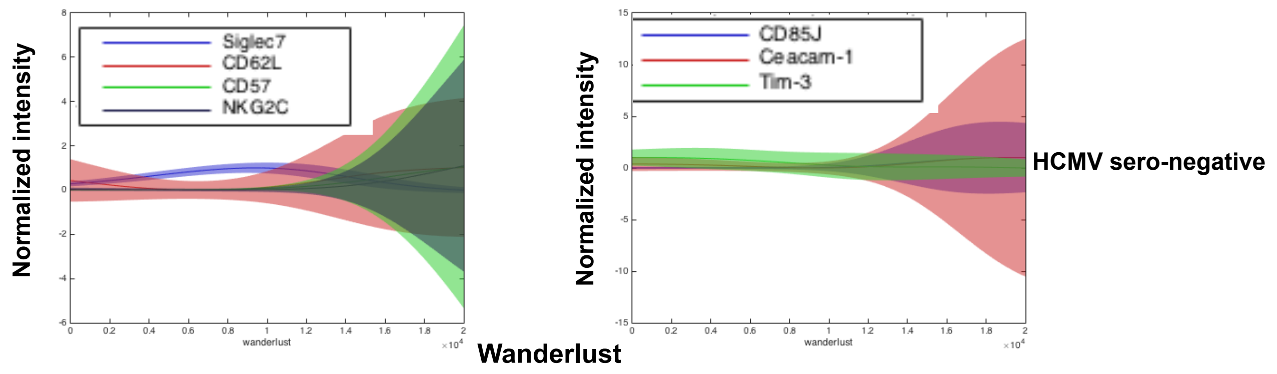

H)

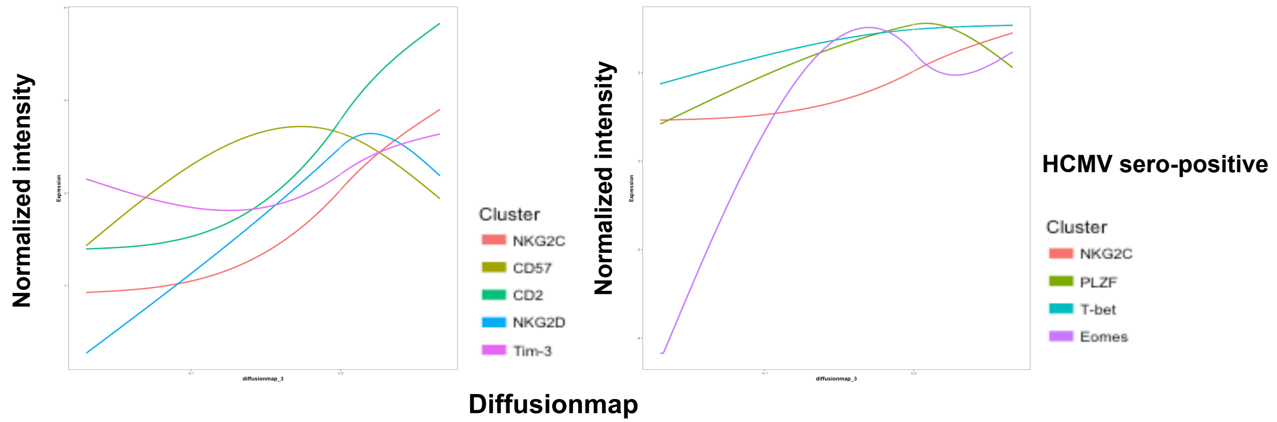

I)

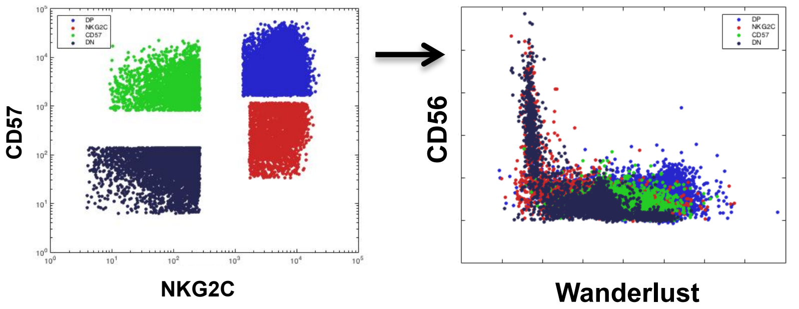

J)

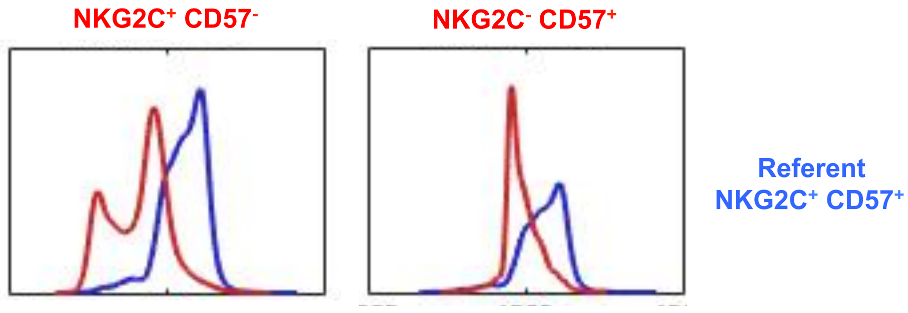

Figure S1

A)

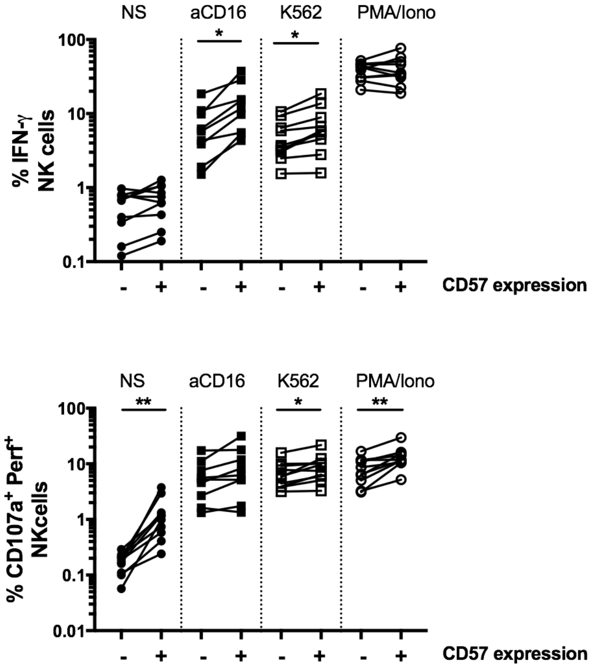
NS
aCD16
K562
PMA/Iono
% IFN-γ NK cells
% TNF-α NK cells
% CD107a+ Perf+ NKcells
% CD107a+ GZB+ NK cells
CD57 expression
100
10
1
0.1
0.01
- +
*
**

Figure S2

B)

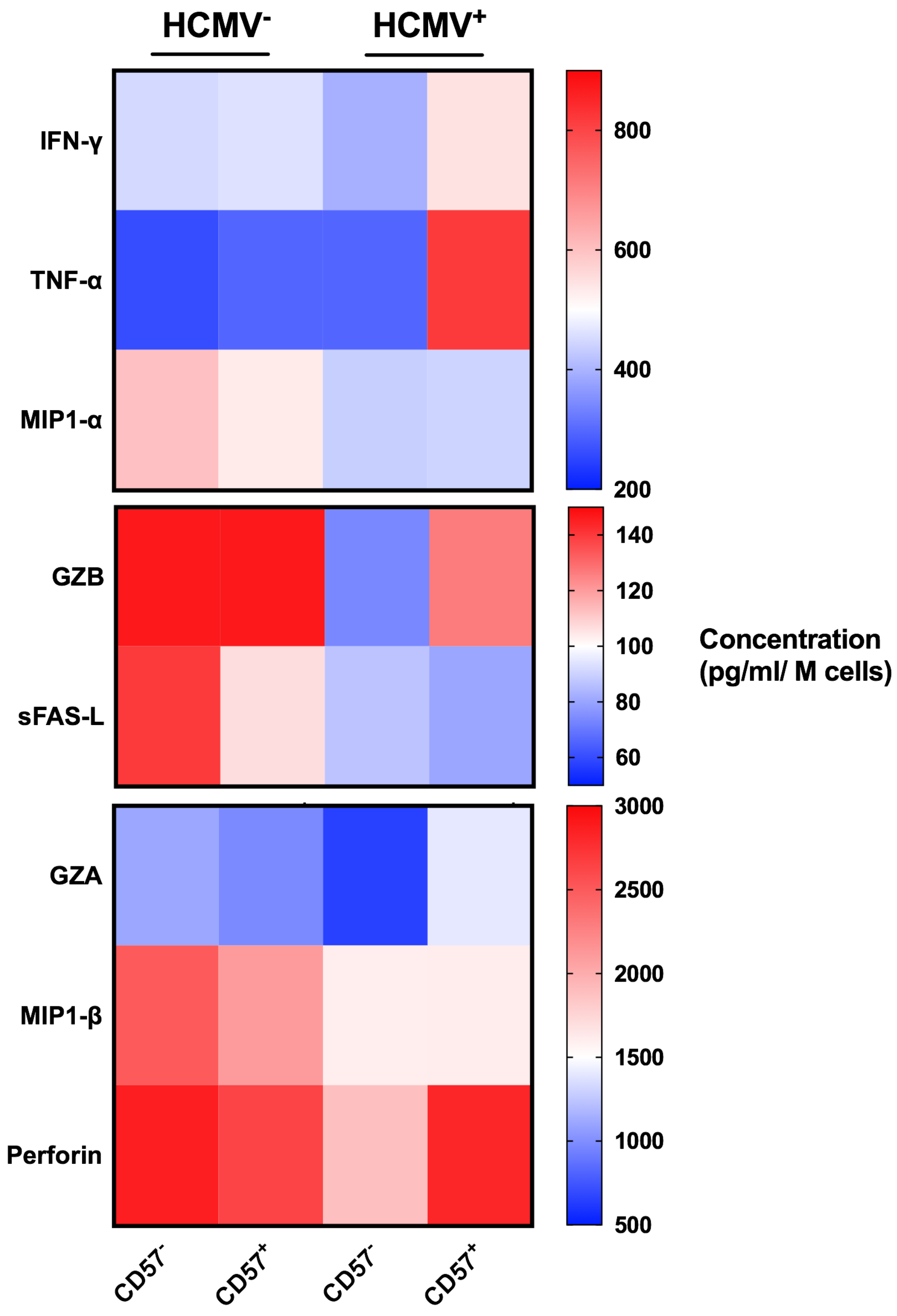

HCMV-
HCMV+
IFN-γ
TNF-α
MIP1-α
GZB
sFAS-L
GZA
MIP1-β
Perforin
CD57-
CD57+
CD57-
CD57+
800
600
400
200
140
120
100
80
60
3000
2500
2000
1500
1000
500
Concentration
(pg/ml/ M cells)

Figure S2

C)

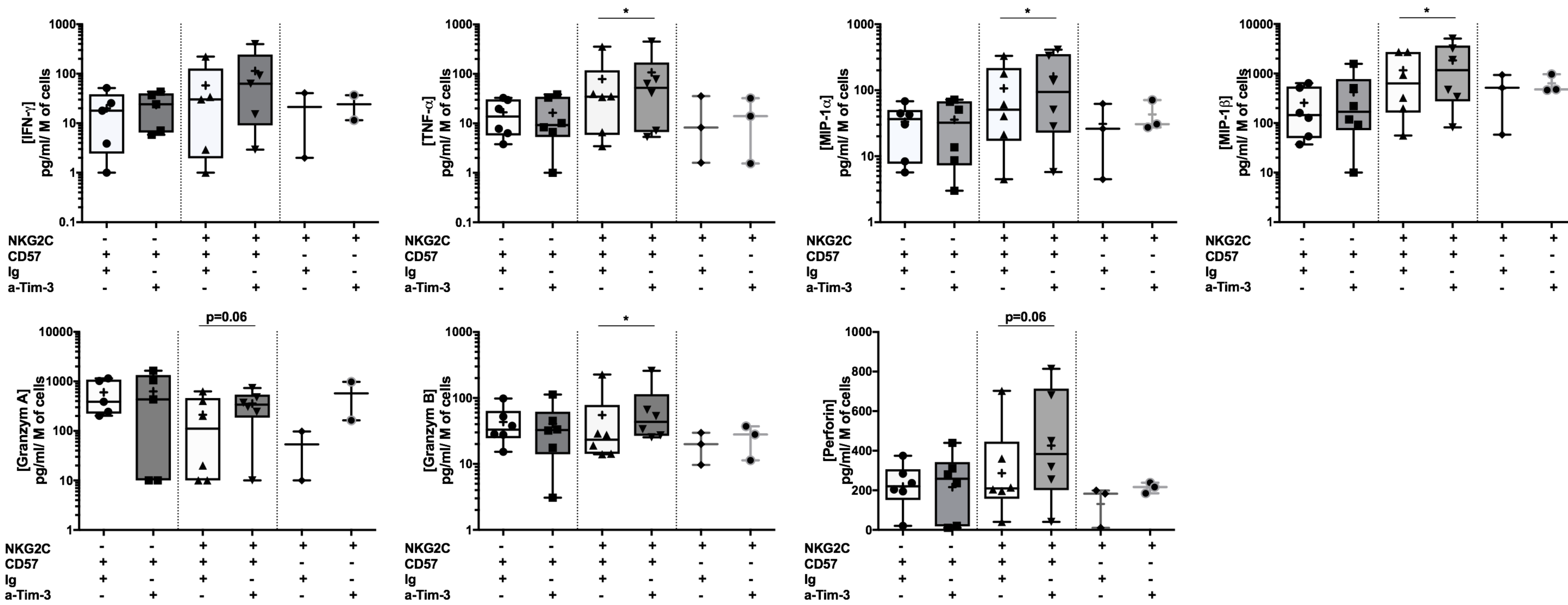
[IFN-γ]
pg/ml/ M of cells
[TNF-α]
pg/ml/ M of cells
[MIP-1α]
pg/ml/ M of cells
[MIP-1β]
pg/ml/ M of cells
[Granzym A]
pg/ml/ M of cells
[Granzym B]
pg/ml/ M of cells
[Perforin]
pg/ml/ M of cells
*
p=0.06
NKG2C - - + + + +
CD57 + + + + - -
Ig + - + - + -
a-Tim-3 - + - + - +

Figure S2

D)

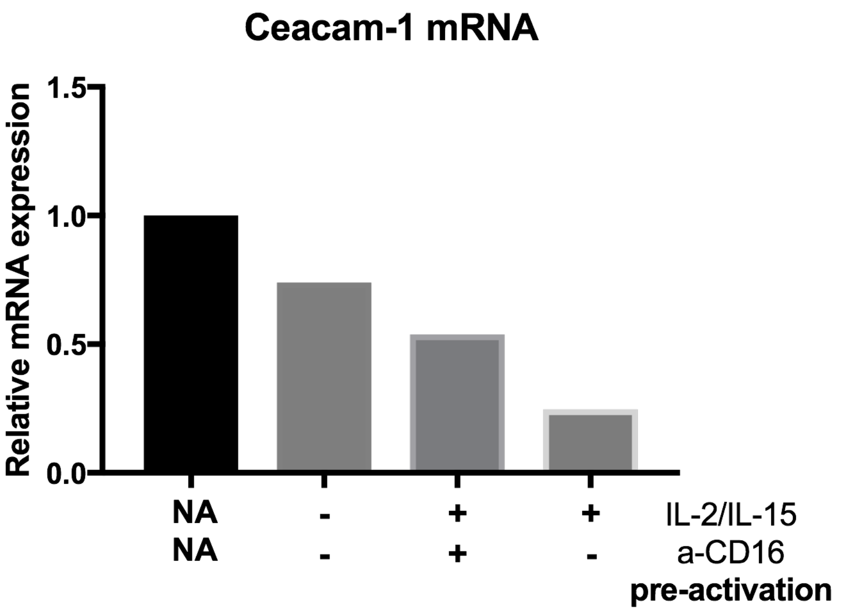

E)

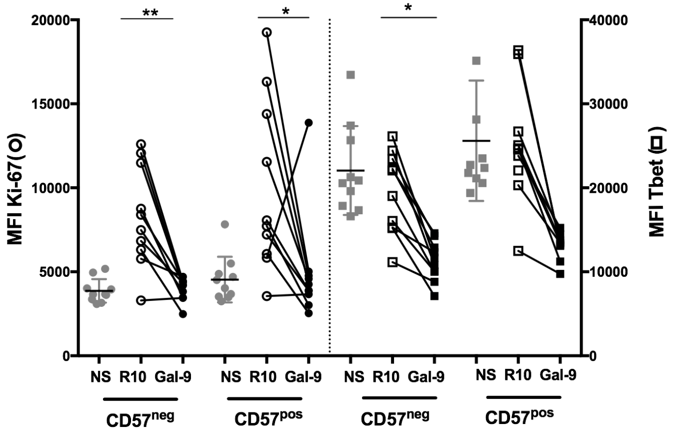

F)

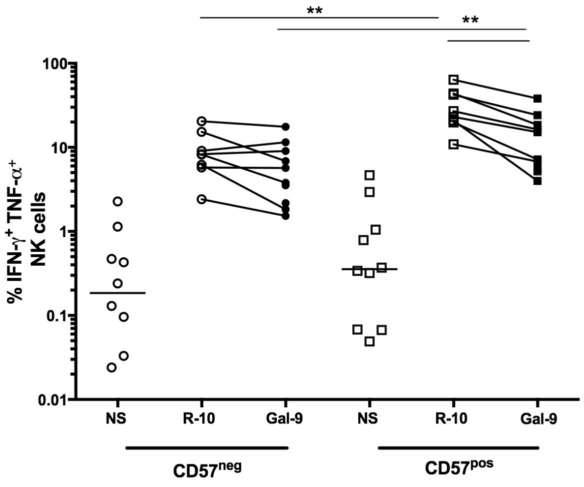

Figure S2

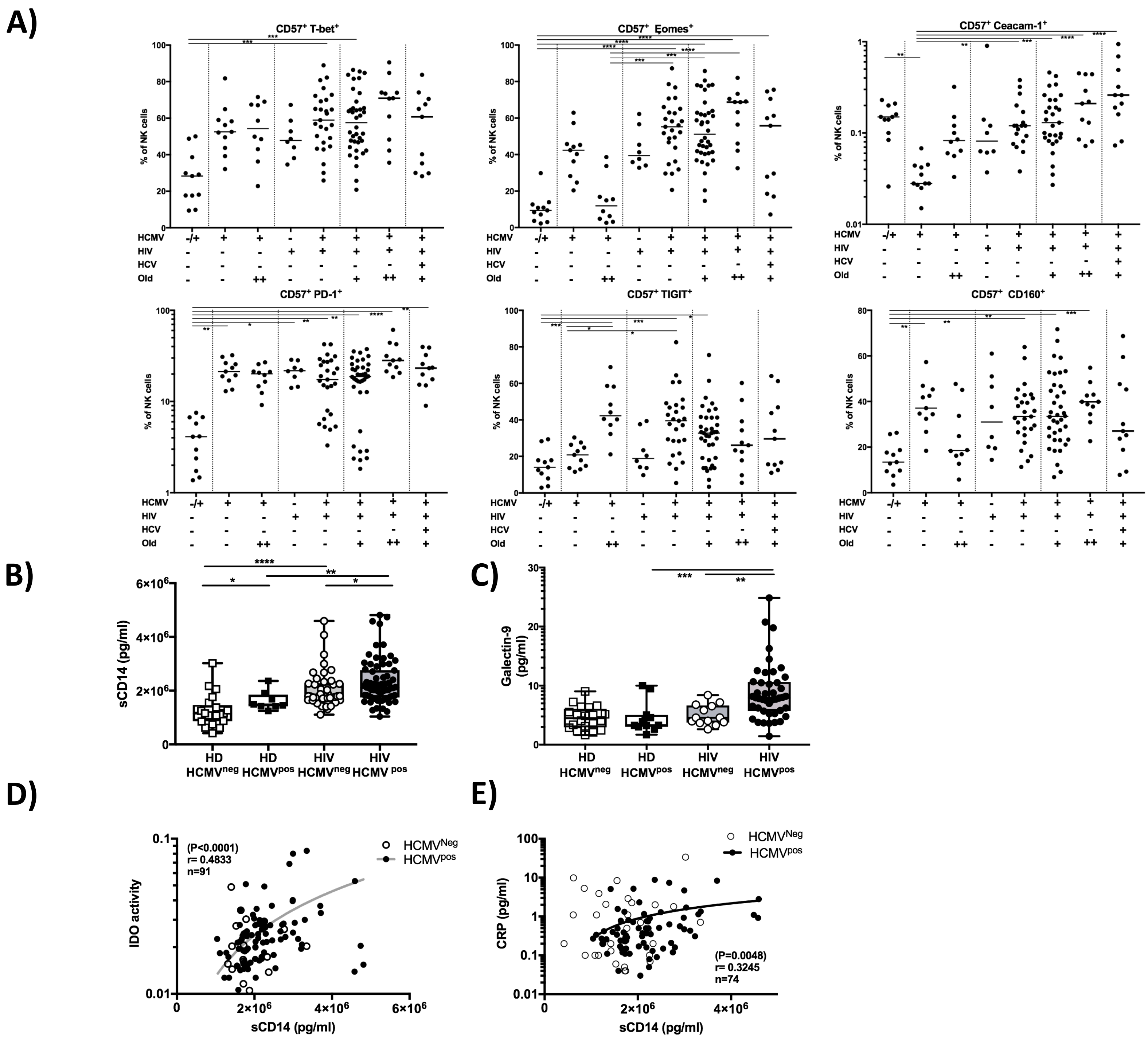
A)
CD57+ T-bet+
CD57+ Eomes+
CD57+ Ceacam-1+
CD57+ PD-1+
CD57+ TIGIT+
CD57+ CD160+
% of NK cells
HCMV
HIV
HCV
Old
B)
sCD14 (pg/ml)
HD HCMVneg
HD HCMVpos
HIV HCMVneg
HIV HCMV pos
C)
Galectin-9 (pg/ml)
D)
IDO activity
(P<0.0001)
r= 0.4833
n=91
HCMVNeg
HCMVpos
E)
CRP (pg/ml)
(P=0.0048)
r= 0.3245
n=74
sCD14 (pg/ml)

Figure S3

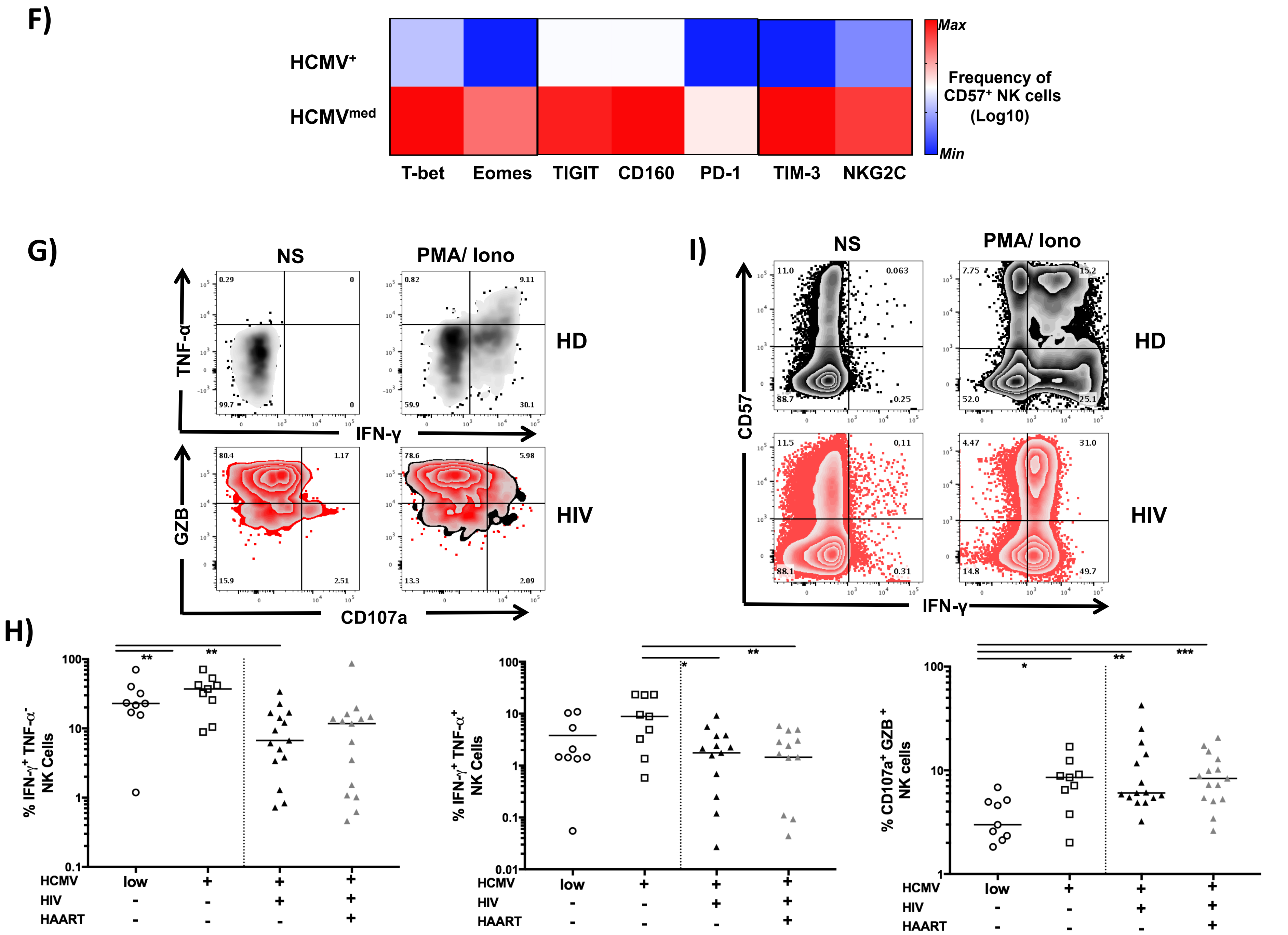
F)
HCMV+
HCMVmed
T-bet
Eomes
TIGIT
CD160
PD-1
TIM-3
NKG2C
Max
Min
Frequency of CD57+ NK cells (Log10)
G)
NS
PMA/ Iono
TNF-α
IFN-γ
HD
0.29
0
99.7
0
0.82
9.11
59.9
30.1
GZB
CD107a
HIV
80.4
1.17
15.9
2.51
78.6
5.98
13.3
2.09
I)
NS
PMA/ Iono
CD57
IFN-γ
HD
11.0
0.063
88.7
0.25
7.75
15.2
52.0
25.1
HIV
11.5
0.11
88.1
0.31
4.47
31.0
14.8
49.7
H)
% IFN-γ+ TNF-α- NK Cells
% IFN-γ+ TNF-α+ NK Cells
% CD107a+ GZB+ NK cells
HCMV low + + +
HIV - - + +
HAART - - - +
*
**
***

Figure S3
