# Supplementary material for: Adaptive NKG2C+CD57+ Natural Killer Cell and Tim-3 Expression During Viral Infections
Source: Front Immunol. 2018 Apr 20;9:686. doi: 10.3389/fimmu.2018.00686 (PMC5919961; doi:10.3389/fimmu.2018.00686)
Supplement: Supplementary file 2 [file data_sheet_2.docx]

**Figure S1: Characterization of NK cells during HCMV infection.**

(A) Heterogeneity of human NK cells during HCMV infection. Frozen PBMCs were surface and intracellular stained for mass cytometry analysis. Samples were barcoded and acquired simultaneously (n=6). t-SNE analysis of 23 parametric data was performed on live CD45^+^CD14^-^CD19^-^CD3^−^CD56^+^ NK cells from three HCMV sero-positive and three HCMV sero-negative donors. Event density in the t-SNE field for all donors compiled according to HCMV sero-status in which a same number of events per donor was included. Normalized protein expression levels for single parameters in t-SNE field were represented in cold-to-hot heat map. Grey lines indicated the border between canonical NK cell populations and adaptive NK cell populations found in HCMV sero-positive donors.

(B) Illustrative phenotype of NK cells from HCMV-seropositive and HCMV sero-negative donor. Cells were gated on live lymphocytes CD45^+^CD14^-^CD19^-^ CD3^−^CD56^+^ NK cells.

(C) Representative phenotype of gated NKG2C^-^ and NKG2C^+^ NK cells from an HCMV-seropositive donor. NK cell subsets were overlaid in order to compare relative proteins expression.

(D) Unbiased clusters analysis of NK cell subsets identified in HCMV sero-negative and sero-positive donors. The median intensity of each protein was normalized and represented by a hot to cold heat map.

(E) Tim-3 and Caeacam-1 expression in CD56^dim^ NK cells during HCMV infection. Illustrative Zebra plot of CD57, Tim-3, NKG2C and Caecam-1 in gated CD56^dim^ NK cells from HCMV seronegative and seropositive donors.

(F) Enhancement of NKG2C and Tim-3 acquisition in CD56^dim^ NK cells by HCMV-specific immune response. Identification of NK cell clusters associated with HCMV infection by flow cytometry. Three-dimensional representation of computational analysis of NK cells from patients with different levels of anti-HCMV IgG. X, Y and Z dimensions were represented by tSNE1, tSNE2 and CD57 (or Tim-3) respectively. A heat-map was included to represent the intensity of NKG2C, Siglec-7 and Ceacam-1 expression in NK cells.

(G) Characterization of NK cell maturation according to wanderlust trajectory in HCMV sero-negative donors. The Wanderlust trajectory is fixed to an arbitrary scale where the most immature NK cells (CD56^bright^) are at 0 and the most mature at 1. The traces demonstrated the relative expression patterns and variation of Siglec-7, CD62L, CD57, NKG2C, CD85j, Ceacam-1, and Tim-3 across human NK cell differentiation.

(H) Characterization of human NK cell’s maturation according to diffusion map in HCMV sero-positive donors (n=12). The traces demonstrated the relative expression patterns and variation of CD57, NKG2C, NKG2D, CD2, Tim-3, PLZF, T-bet and Eomes across differentiation. NKG2A, CD27, CD38, CD161, CX3CR1 and Helios staining were included in the analysis but data are not shown.

(I) Terminal maturation of CD57^+^NKG2C^+^ NK cells according to Wanderlust trajectory. The differentiation of CD57^-^NKG2C^-^, CD57^+^NKG2C^-^, CD57^-^NKG2C^+^, and CD57^+^NKG2C^+^ CD56^dim^ NK cells was evaluated according to their Wanderlust score.

(J) Progressive acquisition of CD57 and NKG2C during NK cell differentiation. CD56^dim^ NK cell subsets were identified according to the expression of CD57 and NKG2C. Wanderlust scores were compared by using CD57^+^NKG2C^+^ NK cells (or CD57^-^NKG2C^-^, data not shown) as reference for NK cell maturation (blue line).

**Figure S2: Regulation of CD57^+^ NK cells functions by the Tim-3 pathway.**

(A) Enhanced effector functions of CD57^+^ NK cells. PBMCs from ten HCMV sero-positive donors were stimulated by CD16 ligation, co-culture with K562 cell lines or PMA/Ionomycin during 5 hours. Effector functions such as IFN-γ or TNF-α secretion, degranulation and cytotoxicity (Perforin, Granzyme B) were assessed by intra-cellular flow cytometry on gated CD3^-^CD56^dim^CD57^-^ and CD3^-^CD56^dim^CD57^+^ NK cells.

(B) Enhanced release of effector molecules by CD57^+^ NK cells during HCMV infection. CD57^-^ and CD57^+^ NK cells were sorted and stimulated by PMA/Ionomycin during 5 hours. Supernatant were collected and analyzed by Luminex.

(C) NKG2C^+^CD57^-^ and NKG2C^-^CD57^+^ NK cells are independent of Tim-3 regulation. Sorted NK cell subsets from HCMV sero-positive donors were pre-incubated with anti-Tim-3 or IgG control before overnight stimulation by CD16 ligation. Supernatants were analyzed by Luminex and the concentrations of each analyte were represented by heat map.

(D) Ceacam-1 silencing in human PBMCs. Three PBMC samples from HCMV sero-positive donors were stimulated overnight with or without cytokines (IL-2 and IL-15) and CD16 ligation before electroporation with *Ceacam-1* or *GAPDH* siRNA. The mRNA of transfected cells were isolated 24 h after incubation at 37°C and 5% CO_2_ and compared to ex vivo mRNA level of non-transfected NK cells in order to measure gene expression silencing by RT-PCR.

(E) Decreased proliferation and T-bet expression in NK cells after CD16 stimulation in presence of Galectin-9. PBMC were stimulated by CD16 ligation in the presence of recombinant Galectin-9 (1 µM).

(F) Reduced cytokines secretion in CD57^+^ NK cells after Tim-3 engagement with Galectin-9. P < 0.05, < 0.01 were considered as significant and represented as (*) and (**) respectively. The Wilcoxon signed rank test was used to compare paired samples.

**Figure S3: Inhibitory receptors and NK cells functions during HIV infection.**

(A) Phenotype of mature CD57^+^ NK cells in HCMV/HIV co-infected patients. Total NK cells were gated from frozen PBMCs of healthy donors and treated HIV-infected patients. The first vertical line distinguished non-infected controls from Singaporean to Malaysian cohorts. HIV-infected individuals were stratified according to their immune response anti-HCMV, HCV co-infection status and age. The co-expression of CD57 with surface and intracellular transcription factors in NK cells was studied by flow cytometry.

(B) Enhanced release of sCD14 and (C) Galectin-9 during HCMV and HIV infection. The levels of these molecules were measured by Elisa in healthy donors or HIV-infected patients. Patients were segregated according to HCMV status (anti HCMV IgG<50 versus IgG=500, median values)

(D-E) Immune activation and microbial translocation during HCMV infection in HIV-infected patients. Plasma samples from non-infected (n=28) and HIV-infected (n=111) individuals were analyzed by LC-MS/MS in order to measure IDO activity (L-kynurenine/Tryptophan ratio). Positive correlations were established between sCD14 and IDO activity (n=91, p<0.0001) or CRP levels (n=74, p=0.0048) in the plasma of treated HCMV/HIV-infected patients. We used the non-parametric Spearman rank-Order correlation test to compare correlation between IDO activity or CRP and sCD14 concentrations in the plasma.

(F) Impairment of HCMV-associated signature on NK cells during HCV/HIV co-infection. The frequencies of inhibitory receptors and transcription factors in mature NK cells (CD57^+^CD56^+^CD3^neg^) from HCV/HIV-coinfected patients were stratified according to anti-HCMV IgG titers. A cold to hot heat map represented the frequencies (Log10) of subsets in each patient group (n=8).

(G-I) Decreased cytokines secretion ability and skewed toward a cytotoxic phenotype of NK cells during HIV infection. PBMCs from healthy control groups, longitudinal follow-up and cross-sectional study cohorts were polyclonally stimulated with PMA/ Ionomycin. Total (G and I) or CD57 gated (H) NK cells were assessed for their degranulation (CD107a), cytotoxicity (Granzyme B) and cytokines secretion abilities. Groups of HIV-infected patients and non-infected controls were analyzed by Mann-Whitney U test to compare values. P < 0.05, < 0.01, <0.001 and p< 0.0001 were considered as significant and represented as (*), (**), (***) and (****) respectively.
