# Supplementary material for: Adaptive NKG2C+CD57+ Natural Killer Cell and Tim-3 Expression During Viral Infections
Source: Front Immunol. 2018 Apr 20;9:686. doi: 10.3389/fimmu.2018.00686 (PMC5919961; doi:10.3389/fimmu.2018.00686)
Supplement: Supplementary file 3 [file data_sheet_3.PDF]

Table S1: List of antibodies for mass cytometry

ANTIGEN	Label (Atomic mass)
CD45	89
CD14	112
CD57	115
HLA-DR	142
CD69	145
CD8	146
CD4	147
CD45RO	148
CD49a	149
KLRG1	150
CD27	151
CD122	152
CD103	153
T-bet	155
Granzyme A	157
CD56	158
CD161	159
NKp44	160
CD38	161
Ki-67	162
CD127	163
Granzyme B	164
IL-18R	165
NKp46	166
Tim-3	167

CD3	168
CD25	169
NKG2C	170
Eomes	171
CD94	172
NKp30	173
CD160	174
Perforin	175
DNA	191/193
Cisplatin	195
CD16	209

Table S2: List of antibodies and relative assays

ANTIGEN	FLUOROCHROME	CLONE	COMPANY	ASSAY
CD3	FITC	UCHT1	BioLegend	Phenotyping<br>Sorting
CD3	APC-Cy7	SK7	BD Biosciences	Functional
CD3	BUV395	UCHT1	BD Biosciences	Phenotyping
CD3	BV786	UCHT1	BD Biosciences	Phenotyping
CD8	BUV805	RPA-T8	BD Biosciences	Phenotyping
CD56	PE-Cy5	HCD56	BioLegend	Phenotyping<br>Functional<br>Sorting
CD56	BUV563	HCD56	BD Biosciences	Phenotyping
CD56	FITC	HCD56	BioLegend	Phenotyping
CD16	AF700	3G8	BioLegend	Phenotyping<br>Functional
CD16	APC-Cy7	3G8	BioLegend	Sorting
CD16	Purified	3G8	BioLegend	Stimulation
CD19	BUV737	SJ25C1	BD Biosciences	Phenotyping
CD62L	APC-Cy7	DREG-56	BioLegend	Phenotyping
CD27	BV650	L128	BD Biosciences	Phenotyping
CD27	BV750	L128	BD Biosciences	Phenotyping
CD57	PB	HCD57	BioLegend	Phenotyping
CD57	BV570	HNK-1	BioLegend	Functional
CD335	APC	9E2	BioLegend	Phenotyping
CD38	PE	HB7	eBioscience	Phenotyping
CD38	BUV737	HB7	eBioscience	Phenotyping
CD94	BUV661	HP-3D9	BD Biosciences	Phenotyping
IFN-γ	BV605	B27	BD Biosciences	Functional
TNF-α	PE-Cy7	Mab11	BioLegend	Functional

Granzyme B	PECF594	GB11	BD Biosciences	Functional
Perforin	BV421	B-D48	BD Biosciences	Functional
CD107a	BV786	H4A3	BD Biosciences	Functional
T-bet	PE	4B10	BioLegend	Functional
T-bet	BV421	4B10	BioLegend	Functional
Ki-67	AF647	Ki-67	BioLegend	Functional
Tim-3	Purified	F38-2E2	BioLegend	TIM-3 blockade
Ig control	Purified	MOPC-21	BioLegend	TIM-3 blockade
Tim-3	BV650	F38-2E2	BioLegend	Phenotyping
Tim-3	A-700	344823	R&D	Phenotyping
Tim-3	PE	344823	R&D	Phenotyping
NKG2C	PE	134591	R&D	Phenotyping
Ceacam-1	BV421	B1.1/CD66	BD Biosciences	Phenotyping
Siglec-7	APC	6-434	BioLegend	Phenotyping
CD85j	PE-Cy7	GHI/75	BioLegend	Phenotyping
Eomes	PE-Cy7	WD1928	eBioscience	Phenotyping
PLZF	APC	6318100	R&D	Phenotyping
Helios	Pe-eF610	22F6	eBioscience	Phenotyping
CD161	PE-Cy5	DX12	BD Biosciences	Phenotyping
NKG2A	Vio-bright FITC	CD159a	Miltenyi	Phenotyping
NKG2D	BV650	1D11	BD Biosciences	Phenotyping
PD-1	FITC	MIH4	BD Biosciences	Phenotyping
CD2	BV711	RPA-2-10	BD Biosciences	Phenotyping
CX3CR1	PerCP/Cy5.5	2A9-1	BioLegend	Phenotyping
HLA-DR	BV605	L243	BioLegend	Sorting

Table S3: Clinical features of HIV patients*

Name of Group	Number of Patients	Age Median (range)	HCMV (IgG titers) Median (range)	CD4 (cells/uL) Median (range)	sCD14(pg/mL) Median	HIV RNA (<50 copies/mL) Number of patients	HIV RNA Blips (range)	HAART	HAART Duration Years (Median)	Smoking Status (Yes/No/NA)	HCV	Gender Nb of Females
**HCMV+/HCV+/HIV+**	11	39 (31-52)	500 (160-500)	533 (93-813)	2.23E+06	10/11	83	Yes	2.74	7/4/0	Yes	0/11
**HCMV-/HIV+/ Young**	15	34 (22-39)	8.66 (0.15-48)	580 (21-1041)	1.72E+07	12/15	(68486-2798000)	Yes	4.75	2/10/6	No	2/15 (13%)
**HCMV+/HIV+/ Young**	28	36 (27-41)	500 (110-500)	516 (0-1230)	1.80E+06	26/28	(159-13210)	Yes	3.92	7/8/13	No	7/28 (25%)
**HCMV+/HIV+/Middle**	37	50 (45-64)	487 (80-500)	575 (117-1367)	2.03E+06	36/37	1060	Yes	8.67	11/18/8	No	6/37 (16.25%)
**HCMV+/HIV+/Old**	12	71 (66-82)	500 (55-500)	550 (250-908)	2.45E+06	12/12	NA	Yes	5.47	1/8/2	No	4/12 (33.33%)
**Longitudinal/ HIV+**	15	40 (31-62)	NA	40 (0-186)	1.83E+06	0/15	(2500-18217150)	No	0	4/6/5	No	3/15 (20%)
**Longitudinal/ HIV+**	15	40 (31-62)	NA	308 (55-515)		13/15	(388-7320)	Yes	0.88	4/6/5	No	
**HIV-/ Young**	16	33 (29-39)	131 (0.15-500)	NA	1.14E+06	NA	NA	NA	NA	3/13/0	NA	2/16 (12.5%)
**HIV-/ Old**	14	51 (46-62)	109 (0.15-500)	NA	1.38E+06	NA	NA	NA	NA	0/14/0	NA	6/14 (42.86%)
